# Preferential Binding of Hot Spot Mutant p53 Proteins to Supercoiled DNA *In Vitro* and in Cells

**DOI:** 10.1371/journal.pone.0059567

**Published:** 2013-03-26

**Authors:** Marie Brázdová, Lucie Navrátilová, Vlastimil Tichý, Kateřina Němcová, Matej Lexa, Roman Hrstka, Petr Pečinka, Matej Adámik, Borivoj Vojtesek, Emil Paleček, Wolfgang Deppert, Miroslav Fojta

**Affiliations:** 1 Department of Biophysical Chemistry and Molecular Oncology, Institute of Biophysics, Academy of Sciences of the Czech Republic, v.v.i., Brno, Czech Republic; 2 Department of Tumor Virology, Heinrich-Pette-Institute, Leibniz Institute for Experimental Virology, Hamburg, Germany; 3 Regional Center for Applied Molecular Oncology, Masaryk Memorial Cancer Institute, Brno, Czech Republic; 4 Faculty of Informatics, Masaryk University, Brno, Czech Republic; 5 Environmental Center, Faculty of Science, University of Ostrava, Ostrava, Czech Republic; 6 Central European Institute of Technology, Masaryk University, Brno, Czech Republic; Virginia Commonwealth University, United States of America

## Abstract

Hot spot mutant p53 (mutp53) proteins exert oncogenic gain-of-function activities. Binding of mutp53 to DNA is assumed to be involved in mutp53-mediated repression or activation of several mutp53 target genes. To investigate the importance of DNA topology on mutp53-DNA recognition *in vitro* and in cells, we analyzed the interaction of seven hot spot mutp53 proteins with topologically different DNA substrates (supercoiled, linear and relaxed) containing and/or lacking mutp53 binding sites (mutp53BS) using a variety of electrophoresis and immunoprecipitation based techniques. All seven hot spot mutp53 proteins (R175H, G245S, R248W, R249S, R273C, R273H and R282W) were found to have retained the ability of wild-type p53 to preferentially bind circular DNA at native negative superhelix density, while linear or relaxed circular DNA was a poor substrate. The preference of mutp53 proteins for supercoiled DNA (supercoil-selective binding) was further substantiated by competition experiments with linear DNA or relaxed DNA *in vitro* and *ex vivo*. Using chromatin immunoprecipitation, the preferential binding of mutp53 to a sc mutp53BS was detected also in cells. Furthermore, we have shown by luciferase reporter assay that the DNA topology influences p53 regulation of BAX and MSP/MST1 promoters. Possible modes of mutp53 binding to topologically constrained DNA substrates and their biological consequences are discussed.

## Introduction

Inactivation of the *TP53* gene by point mutations is a common event in human cancers (about 50% of all malignancies hold a mutated *p53* locus) [1]. Mutant p53 (mutp53) is connected with cancer development and progression, as some point mutations not only abrogate cardinal tumor suppressor functions of p53 in cell-cycle arrest, DNA repair and apoptosis, but also confer new oncogenic functions to mutp53 (“gain-of-function”, mutp53GOF).

The p53 protein displays classical features of a sequence-specific transcriptional factor, including a transactivation domain, a sequence-specific DNA binding core domain (aa ∼100–∼300, p53CD) that plays a crucial role in recognition of the p53 target sites (p53CON), and an oligomerization domain (aa ∼325–∼356). In addition, p53 is unique due to a second autonomous DNA binding site at the extreme C-terminus (C-terminal DNA binding site, CTDBS, aa 363–382) [2]. The basic CTDBS, which has been shown to possess non-sequence-specific nucleic acid binding ability ([3], reviewed in [4]), plays a crucial role in processes of (i) DNA repair, (ii) DNA recombination and in (iii) transactivation of downstream promoters *in vivo* [5], [6], [7]. The C-terminal domain is also responsible for the fast sliding of p53 along DNA [8]. In contrast to many other transcription factors, wild-type p53 (wtp53) binds preferentially to genomic regions with high DNA-encoded nucleosome occupancy [9].

**Figure 1:**
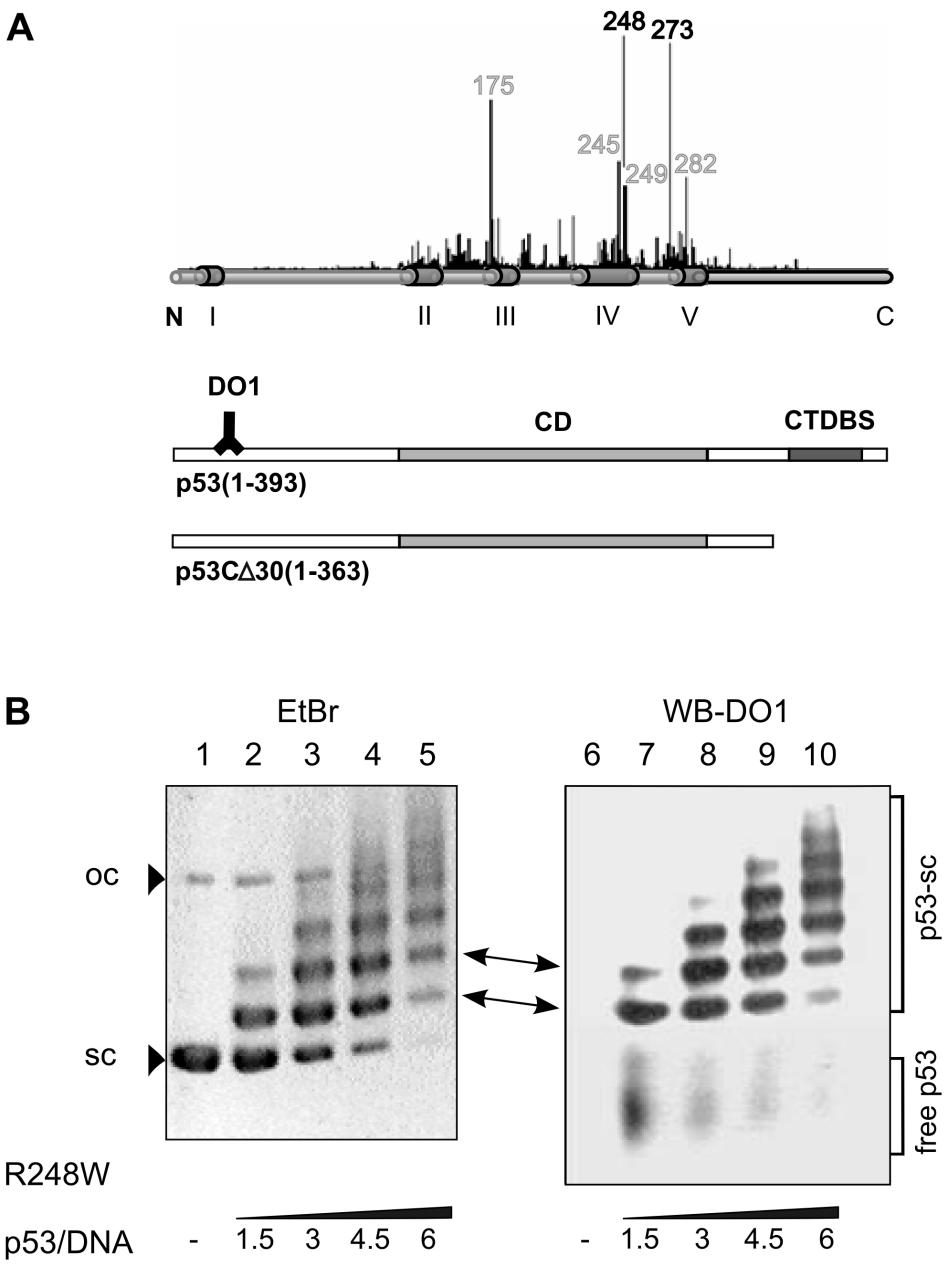
Hot spot mutp53 proteins and R248W preferential binding to scDNA. A) Distribution of hot spot mutations on full length p53 (conserved regions marked by cylinders) with a bar code representing their frequency; scheme of p53 molecules (p53 (aa 1–393), p53CΔ30 (aa 1–363)), DNA binding domains (shaded, central (CD) and C-terminal (CTDBS)) and positions of mAb epitope of DO1 (aa 21–25) are marked. The conformation of mutp53 proteins is labeled: grey (conformation mutants) and black (contact mutants). B) ScDNA binding of p53 R248W by agarose electrophoresis and DO1 immunoblotting. Increasing amounts of p53 protein (marked by the p53/DNA molar ratio) (lanes 2–5) were incubated with scDNA (pBSK, 200 ng) for 20 min and then separated in a 1% agarose gel at 4°C. DO1 immunodetection of R248W binding to scDNA showed that each retarded band of Et-Br visualized DNA on the agarose gel (lanes 2–5) corresponds to a p53 band on the DO1 immunoblot (lanes 7–10). Open circle DNA (oc) was not bound by p53.

More than 80% of the known cancer-associated p53 mutations are located within the core domain, where six hot spots (Arg-175, Gly-245, Arg-248, Arg-249, Arg-273 and Arg-282) represent about 40% of all p53 mutations [10]. The basic categorization of p53 mutations takes into account the impact of the mutation on protein structure/stabilization and on its interaction with DNA (Fig. 1A): (a) type I, DNA contact sites such as Trp-248 (minor groove) and His-273 (phosphate backbone); and (b) type II, mutations that destabilize the protein conformation with minor distortion (such as Gly-245 and Arg-249) and global denaturation (such as Arg-175 and Arg-282) [11]. Mutp53 proteins are often less degraded in contrast to wtp53 and thus overexpressed in tumor cells, mainly due to their inability to effectively activate MDM2 [12]. Binding of molecular chaperons (including Hsp70, Hsp90 and CHIP) also contributes to their stabilization [13], [14]. The fact that tumor-derived mutations are localized almost exclusively within the DNA binding core domain underscores the importance of DNA recognition for p53 function. Certain mutp53 proteins are inherently capable to bind p53CONs (within 50-mer oligonucleotides) [15] at sub-physiological temperatures (20° or 25°C, when all hot spot mutant p53 core domains are folded [11]). Additionally, a stabilization of mutp53-DNA binding was demonstrated by C-terminal or p53CD modification. Also the presentation of p53CON in non-B DNA conformation (such as stem-loop DNA structures) had a dramatically positive influence on the recognition of such target sites by some mutp53 proteins [16]. Mutp53 proteins have retained selectivity for *in vitro* recognition of certain non-B DNA structures. For example, four-way junctions, hairpins, G-quadruplexes and structures formed by CTG.CAG trinucleotide repeats are strongly bound by G245S [16], [17], [18]. Furthermore, mutp53 proteins have maintained the ability to strongly bind genomic DNA elements representing matrix attachment regions (MARs), known to exhibit a high potential of base unpairing and presentation of non-B DNA [19], [20], [21], [22]. Recently, we have shown preferential binding of R273H to G/C-rich DNA around transcription start sites in U251 cells [17].

Insensitivity to drugs, resistance to apoptosis, enhanced cell proliferation and/or migration, increased chromosomal instability and nonhomologous recombination are attributed to all hot spot mutp53 proteins, such as mutp53GOFs. Proposed multiple mechanisms include transcriptional and nontranscriptional activities: a) physical interaction with p53 family members p63 and p73; b) interaction with and recruitment by other transcription factors to their consensus binding sites (e.g. Sp1, NF-Y, E2F1, VDR and SREBP-2); c) physical interaction with other cellular proteins (topoisomerase I, MRE1, Pin1, PML, MBP1, p38, p42) and d) direct interaction with structure-specific (non-B DNA secondary structures) and sequence-specific DNA elements or chromatin landscape [23], [24], [25], [26], [27]. Mutp53-DNA binding, direct or indirect, is connected with transactivation or transrepression of many genes (e.g. *MSP/MST1* [28], *CD95/Fas/Apo1* [29], *ID2* [30], *EGR1* [31], *ID4* [32], reviewed in [24]). DNA fragments detected by mutp53 specific chromatin immunoprecipitation are marked as mutp53 binding sites (mutp53BS). Recruitment of mutp53 to such sites (e.g. in promoters of regulated genes) through protein-protein interaction is currently preferred model of interaction of mutp53 with DNA in cells. However, our recent study rather argue for at least Sp1 and ETS1-independent binding of mutp53 (R273H) to G/C rich DNA in U251 cells [17].

Formation of non-B DNA secondary structures is facilitated under favorable conditions (e.g. negative supercoiling and stabilization by bound protein) [33] during DNA transcription, replication and other processes in cells [34]. Subsequently, it may propagate across large distances through the genome due to the dynamic nature of DNA-nucleosome interactions [35].

Our previous studies had focused on the investigation of wtp53 *in vitro* binding to supercoiled plasmid DNA (scDNA) as a DNA substrate mimicking some conformational and topological features of DNA in cells. It was shown [36], [37], [38], [39] that wtp53 protein binds preferentially to negatively and positively supercoiled DNAs, both containing and lacking a p53CON sequence. A critical role of the p53 CTDBS in the highly selective recognition of scDNA (supercoil-selective binding, SCS-binding) has been reported [40], [41]. More recently, we have shown that DNA supercoiling enhances sequence-specific DNA binding of wtp53 through modulating non-B DNA structures within internally symmetrical p53 target sites [42], [43].

In this study, we have analyzed for the first time the interaction of seven hot spot mutp53 proteins (R175H, G245S, R248W, R249S, R273H, R273C and R282W) with supercoiled, linear and relaxed circular DNA of plasmids lacking or containing p53CON or mutp53 binding sites (detected by chromatin immunoprecipitation (ChIP)). SCS-binding of mutp53 proteins has been tested *in vitro* in detail using purified mutp53 proteins (full length and C-terminal deletion forms), extracts from cancer cell lines and in cells by ChIP. Similarly to wtp53, we observed mutp53 preference for scDNA with more negative superhelix density. In order to confirm these phenomena in cells, we analyzed mutp53 dependent repression of selected target genes in H1299 and Saos2. We demonstrated that DNA supercoiling strongly enhanced the level of mutp53-mediated repression of the BAX and MSP/MST1 promoters.

## Materials and Methods

### Recombinant Plasmids

Plasmids (pT7-7) encoding full length human wtp53 (aa 1–393, p53fl) and CΔ30-p53 (aa 1–363) were kindly provided by C. Midgley [44]. Plasmids pGEX-2TK encoding GST-p53CD protein (aa 92–312, provided by C. Klein [45], Roche Diagnostics GmbH) and GST-Sp1 (aa 83–621, Sp1 without DNA binding domain [46], Addgene) were used. Mutant p53 forms (R175H, G245S, R248W, R249S, R273H, R273C and R282W) of p53fl and CΔ30-p53 proteins were cloned to pT7-7p53 vectors by core domain substitution in VanD21 restriction sites of the eukaryotic vector pCDNA3.1, kindly provided by R.W. deVereWhite [47]. Supercoiled plasmids pBluescript SK II- (pBSK, Stratagene), pPGM1 (containing p53CON: AGACATGCCTAGACATGCCT [38]), pMSP (containing mutp53 recognition site, 161 bp fragment of MSP/MST1 promoter [28]) were isolated from bacterial strain TOP10 (Stratagene) as described in the Qiagen protocol (Qiagen, Germany). MSP/MST1 fragment was amplified by PCR (MSPF: CTCACTGATGTGTAGCGGTGCT, MSPR: TGTCCAACAGAGTAACCATTAGCC) and cloned into the EcoRV site in pBSK. Plasmids pAA3, pAB10, pAA12 are derivates of the pCRII plasmid containing repetitive sequences - mutp53BSs (Tab. S1) described in [19]. *SmaI* restriction enzyme (Takara) was used for linearization of pBSK (linBSK), pPGM1 (linPGM1) and pMSP (linMSP); similarly *ScaI* (Takara) was used for linearization of pCRII based plasmid DNAs. Relaxed DNA (relDNA) was prepared with topoisomerase I (Takara) according to a protocol described previously [48].

### Purification of p53 Recombinant Proteins

The p53 proteins were purified according to a protocol described previously [19], [40], [49] with some minor modifications. The purity and appropriate size of each protein was analyzed by Coomassie blue staining of 12.5% SDS-PAGE gels (Fig. S1) and intact N-terminal and C-terminal parts were detected by Western blotting (not shown). Mouse monoclonal anti-p53 antibodies (mAb) (DO1 (aa 20–25), Bp53 10.1 (aa 375–379), PAb421 (aa 371–380) and ICA9 (aa 388–393)) and anti-GST Ab (G1160, Sigma) were used. Purification of GST-p53CD protein [45] and GST-Sp1 (aa 83–621, Sp1 without DNA binding domain [46]) was according to a protocol described in [49].

### DNA Binding Assays by Electrophoretic Mobility-shift Assay (EMSA) in Agarose Gels

ScDNA (pBSK, pMSP, pAA3, pAA12, pAB10) or linDNA (linBSK, linPGM1, linAB10 or linMSP) were mixed with p53 proteins at p53 tetramer/DNA molar ratios between 0.25–20 and incubated in binding buffer (5 mM Tris-HCl pH 7.6, 0.5 mM EDTA, 0.01% Triton X-100 and 50 or 150 mM KCl) for 30 min either on ice or 25°C to reach equilibrium as described previously [37]. After 5 h electrophoresis (at 4–6 V/cm), gels were stained with ethidium bromide (Et-Br) for 45 min and photographed using Herolab documentation system (Herolab). The graphs of p53 binding to sc and linDNA were plotted on the basis of Et-Br stained agarose gels. Intensities of bands of free DNA substrates were quantified by IMAGE-QUANT software. Mean values of three independent experiments were plotted in the graph. Graphs show the evaluation of p53-DNA binding as the dependence of % of bound DNA (axis *y*) on the amount of p53 proteins (expressed by molar ratio p53/DNA, axis *x*). Mean values of three independent experiments were plotted in the graph.

### Sc/lin Competition Assays by EMSA

A mix of scDNA (native superhelix density) and linDNA in equimolar ratio were incubated with p53 proteins at p53 tetramer/DNA molar ratios between 1–20 in binding buffer for 30 min either on ice or 25°C to reach equilibrium as described previously [37], [40]. Samples were loaded onto a 1.3% agarose gel containing 0.33x Tris-borate-EDTA (TBE) buffer; in this buffer system linDNA migrates faster than scDNA [40]. After 8 h electrophoresis (at 4–6 V/cm), gels were documented by standard procedure, see above.

### Supershift EMSA Experiment

P53 proteins were preincubated with affinity purified mouse mAbs (50–300 ng) at 25°C for 20 min and incubation continued with addition of mixture of sc/linDNA (total 400 ng) or scDNA (200 ng) for 15 min on ice or at 25°C [50]. Samples were separated on 1% or 1.3% agarose gel in the same conditions as describe above.

### Analysis of p53-DNA Complexes by Immunoblotting

Proteins from agarose gels were blotted onto a nitrocellulose transfer membrane (Protran R; Schleicher and Schuell, Germany) and p53 was detected by primary mouse monoclonal antibody DO1. Details of the procedures are described in [51].

### Immunoprecipitation Assay of p53-DNA Binding at Magnetic Beads (MBIP Assay)

The Ab-p53-DNA complexes were prepared by mixing the DO1 or anti-GST antibody (400 ng) with the purified protein (50 ng) or whole cell lysate (15–30 µg) in binding buffer (50 mM KCl, 5 mM Tris pH 7.6 and 0.01% Triton X-100), followed by 20 min incubation on ice. Then, 300 ng of scDNA (pMSP) and/or the same amount of the linDNAs were mixed with the given immune complexes and incubated in the binding buffer for 30 min on ice. Magnetic beads (15 µl of suspension per sample) coated with protein G (DBG, Dynal/Invitrogen), were added to Ab-p53-DNA complexes after washing in binding buffer and incubated with the beads for 30 min at 10°C while shaking mildly. Beads were separated from the assay using a magnetic particle concentrator. Finally, after triplicate washing in binding buffer, the DNA was released from the beads by heating at 65°C in 15 µl of 1.0% SDS for 5 min and analyzed by agarose gel electrophoresis. The electrophoresis conditions (1% agarose gels containing 1×Tris/Acetate/EDTA buffer, pH 7.9, RT, at 4–6 V/cm) were used for separation of bound DNA. In these conditions scDNA migrates faster than linDNA.

### Human Cell Lines, Transfections and Luciferase Assays

Human Saos2 (HTB-85, ATCC) osteosarcoma cells and the human non-small lung carcinoma cell line H1299 (NCI-H1299, ATCC), both p53 null cell lines, were grown in DMEM medium supplemented with 5% FBS and penicillin/streptomycin (Gibco). All cultures were incubated at 37°C with 5% CO_2_. The luciferase reporter construct containing the mutp53 recognition site MSP/MST1 [28] was constructed by inserting the double digested XhoI/SmaI fragment of the pMSP plasmid into the pGL3-promoter backbone (Promega). The luciferase reporter constructs pGL3-BAX (BAX promoter region −248/−610 bp from transcription start, [52]), pGL3-MDM2-APP [18], pGL3-AA12 (333 bp chip fragment in SmaI/XhoI in pGL3-basic, [19], Tab. S1) and pGL3-basic and pGL3-promoter (Promega) were used. pRL-SV40, a reporter plasmid encoding the Renilla reniformis luciferase was used as a control of transfection efficiency. Saos2 and H1299 cells were seeded in 24-well plates 24 h before transfection. Cells were transfected using Effectine (Qiagen) or Lipofectamine (Invitrogene) according to the manufacturer’s instructions at 80% confluence. When appropriate, 50–100 ng of the p53 expression vector based on pCDNA3.1 (kindly provided by R.W. deVereWhite [47]) or empty vector pCDNA3.1 was co-transfected with 200 ng of reporter construct in supercoiled or relaxed form. About 16–20 h after transfection, extracts were prepared using the Dual Luciferase Assay System (Promega) following the manufacturer’s protocol and luciferase activities were measured in a plate reader luminometer IMMUNOTECH LMT01 (Beckmann). For each construct, relative luciferase activity is defined as the mean value of the firefly luciferase/Renilla luciferase ratios obtained from at least three independent experiments. DNA transfection efficiency of sc and relpGL3 constructs was checked by PCR analysis of transfected reporter vectors after isolation of DNA from fraction of lysates (Fig. S6A).

### Establishing Inducible TO Cell Line

T-Rex is a Tet-regulated mammalian expression system based on the binding of tetracycline or doxocycline to a Tet repressor (TR) resulting in promoter activation of the appropriate Tet-inducible gene [53]. H1299 cells were co-transfected with pcDNA6/TR (encoding TR) and pcDNA4/TO vector (containing mutp53 273H); Blasticidin- and Zeocin-resistant clones were selected after 2 weeks. The experiments involved a clone expressing R273H protein only in the presence of doxocycline (1 µg/ml).

### ChIP Assay

H1299 cells (10 cm dish) transfected with plasmids pGL3-MSP and pMSP (2 µg of each) in sc and lin forms and p53 expression vector (pCDNA3.1; 2 µg) were crosslinked with formaldehyde and subjected to chromatin immunoprecipitation (ChiP) assays as previously described with the following modifications [19]: the sonication of cells was limited to 4 kJ (small probe, Sonoplus Bandelin). Purified antibodies DO1 and IgG were incubated overnight with diluted chromatin and immunoprecipitations were performed with protein G-magnetic beads (Invitrogen). The PCR was performed using the primers targeting MSP/MST1 site in pGL3-MSP (GL2: CTTTATGTTTTTGGCGTCTTCC; RV3: CTA GCAAAATAGGCTGTCCC) and primers targeting MSP/MST1 site in pMSP plasmid (BT7: GCGCGTAATACGACTCACTA and MSP: CTCACTGATGTGTAGCGGTGCT). For quantitative analysis, PCR was carried out for 25 cycles.

### Expression Analysis

H1299 and Saos2 cells (2×10^5^) were transfected using Effectine (Qiagen) or Lipofectamine (Invitrogene) according to the manufacturer’s instructions at 80% confluence. When appropriate, 500 ng of the p53 expression vector or empty vector pCDNA3.1 were used. In the stress condition cells were exposed to 0.1 µM doxorubicin for 16 h [54]. For qRT-PCR analysis, total RNA was isolated by applying NucleoSpin RNA II (MachereyNagel - according to the manufacturés instruction) and 2 µg of RNA were reverse transcribed by the High Capacity RT kit (Applied Biosystems - according to manufacturés protocol). PCR was performed using the Power SYBR Green PCR Master Mix (Applied Biosystems) and EvaGreen (Solis Biodyne) in the standard program (15 min 95°C; 15 s 95°C, 30 s 60°C, 20 s 72°C, 10 s 74°C; 45 cycles) running in an RotorGene 6000 (Corbett Research) and 7900HT Fast Real-Time PCR system (Applied Biosystems). PCR reactions for each sample were repeated in triplicates. The housekeeping genes (HPRT1, GAPDH, Actin) were used as endogenous controls. Relative quantitation of transcript levels with respect to the calibrator (H1299 with empty vector) was done based on 2^−ΔΔCT^ algorithm. The following primer sets were used:

**BAX-F** (GCCCTTTTGCTTCAGGGTTT)+**BAX-R** (TCCAATGTCCAGCCCATGAT)

**GAPDH-R** (AAGGTCATCCCTGAGCTGAA)+**GAPDH-R** (CCCCTCTTCAAGGGGTCTAC)

**Q-MSP-F** (ACAAGCCGCAGTTCACGTTT)+**Q-MSP-R** (TCTCCTCCAGTTGTGCATGC)

**HPRT1-Q1** (TGACACTGGCAAAACAATGCA)+**HPRT1-Q2** (GGTCCTTTTCACCAGCAAGCT).

## Results

### Tumor-associated Mutant p53 Proteins Bind to Supercoiled DNA

**Figure 2:**
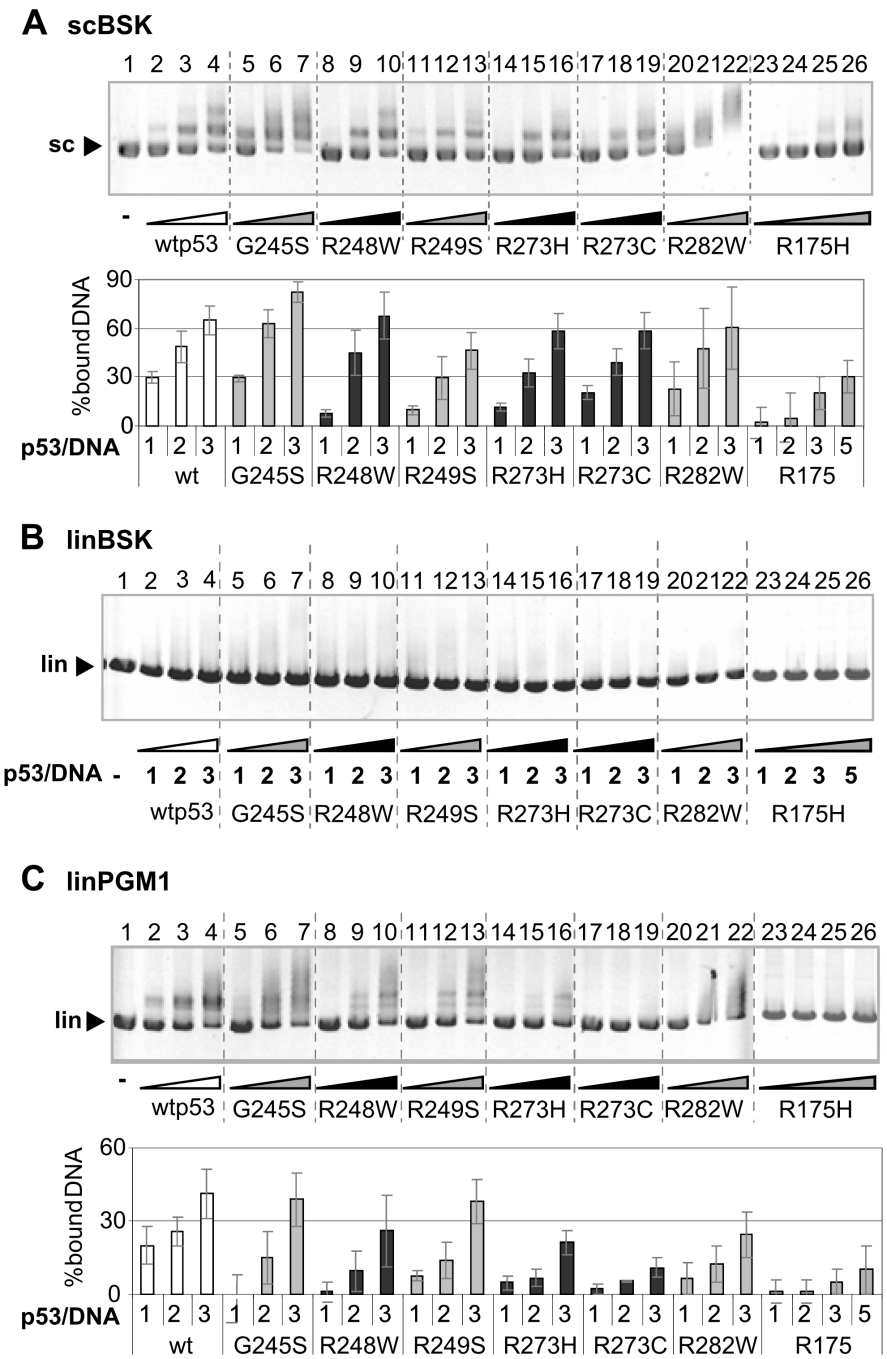
Comparison of mutp53 binding to scDNA and linDNA by EMSA. Mutp53 proteins R175H, G245S, R248W, R249S, R273H, R273C, R282W and wtp53 were bound to scDNA (pBSK, 200 ng, A), linDNA (linBSK, 200 ng, B) and to linDNA with p53CON sequence (linPGM1, 200 ng, C) in p53/DNA molar ratios 1, 2, 3 and 5 (for R175H only) at 25°C; EMSA was performed at 4°C. Column graphs below (pictures A and C) were plotted on the basis of Et-Br stained agarose gels (A and C from three independent experiments); free DNA substrates labeled with arrows were measured by densitometry. Graphs show the evaluation of p53-DNA binding as the dependence of % of bound DNA (axis *y*) on the amount of input of p53 proteins in the reaction (expressed by molar ratio p53/DNA, axis *x*). Bound DNA (%) were calculated as % of decrease of free DNA after binding of p53 in comparison to input DNA (lane 1, 0% of bound DNA). The conformation of p53 proteins is labeled: white (wtp53), grey (conformation mutants) and black (contact mutants).

In order to analyze the DNA binding properties of mutant p53 proteins, we investigated their interaction with scDNA at native superhelix density (on average 15–20 superhelical turns) by agarose gel electrophoresis and immunoblotting techniques. Full length hot spot mutp53 proteins (R175H, G245S, R248W, R249S, R273C, R273H and R282W, Fig. 1A) were bound to scDNA lacking the p53 recognition sequence (scBSK). Similarly as observed previously with wtp53 [38], binding of full length mutp53 proteins to scDNA resulted in ladders of retarded scDNA bands on the agarose gel (Fig. 1B shows an example for R248W). Individual bands in the ladders differed in the number of p53 tetramers bound per scDNA molecule (p53/DNA), as deduced from the comparison of the relative intensities of Et-Br stained DNA bands with the protein bands on the corresponding immunoblot (Fig. 1B) [51]. All seven hot spot mutp53 proteins were analyzed in detail at molar ratios p53/DNA = 1 to 5 (Fig. 2A). To quantify differences between mutp53 proteins, DNA binding was evaluated by densitometry of the band corresponding to free (protein-unbound) DNA. From the relative decrease of the band intensity, the fraction of DNA bound by the protein was calculated and plotted in the graphs shown in Fig. 2A (average of at least 3 independent experiments), demonstrating differences in the affinities of mutp53 proteins to scBSK. Mutants R248W, R249S, R273H, R273C and R282W (Fig. 2A, lanes 8–10, 11–13, 14–16, 17–19, 20–22) showed a similar extent of binding (suggesting similar affinity) to scDNA as wtp53 (Fig. 2A, lanes 2–4). For G245S (Fig. 2A, lanes 5–7) proteins binding to scDNA was slightly stronger compared to the former mutp53 proteins. Interestingly, we observed a weaker binding to DNA for R175H (lanes 23–26) as compared to the other mutp53 proteins (higher p53/scDNA ratios were required to obtain similar scDNA retardation). Taken together, these data imply that p53-scDNA binding, measured as stable nucleoprotein complex formation by EMSA, is a common property of both wtp53 and all tested mutp53 proteins (despite certain differences in their affinities).

### Effect of DNA Topology on Mutp53 DNA Recognition

To demonstrate the effect of DNA topology on DNA recognition by mutp53 proteins, binding of individual proteins to scDNA (scBSK, Fig. 2A) was compared with binding of the same mutants to linear DNAs lacking (linBSK, Fig. 2B) or containing a wtp53 recognition site (p53CON; linPGM1, Fig. 2C).

The linBSK fragment was bound by wtp53 as well as by all mutp53 proteins with lower affinities than scBSK (Fig. 2A, B and Fig. S2A–C). Nearly no binding was observed at low molar ratios p53/DNA (up to 3, Fig. 2B, Fig. S2A–C). For long (3 kbp) linear DNA containing p53CON sequences (linPGM1; p53CON AGACATGCCTAGACATGCCT [38]), a higher extent of binding (compared to linBSK) was observed for G245S, R248W, R249S and R273H at molar ratios as low as 2 (Fig. 2C). These mutants thus have retained a certain ability to recognize the p53CON site within long linDNA. However, the retarded bands of mutp53-linPGM1 appeared as smears, suggesting a lower stability of the mutp53-linPGM1 complexes and their dissociation during electrophoresis, in contrast to wtp53 binding to linPGM1 producing sharp, distinct bands and exhibiting the highest binding affinity (at molar ratio 1, Fig. 2C, lane 2). These binding studies showed that all p53 proteins (both wt and mut) bound scDNA with a considerably higher affinity compared to binding of linDNA lacking p53CON and that mutp53 proteins bound more efficiently to scDNA than to p53CON within linDNA (Fig. 2C).

### Supercoil-selective Binding is a Common Feature of p53 Hot Spot Mutants

**Figure 3:**
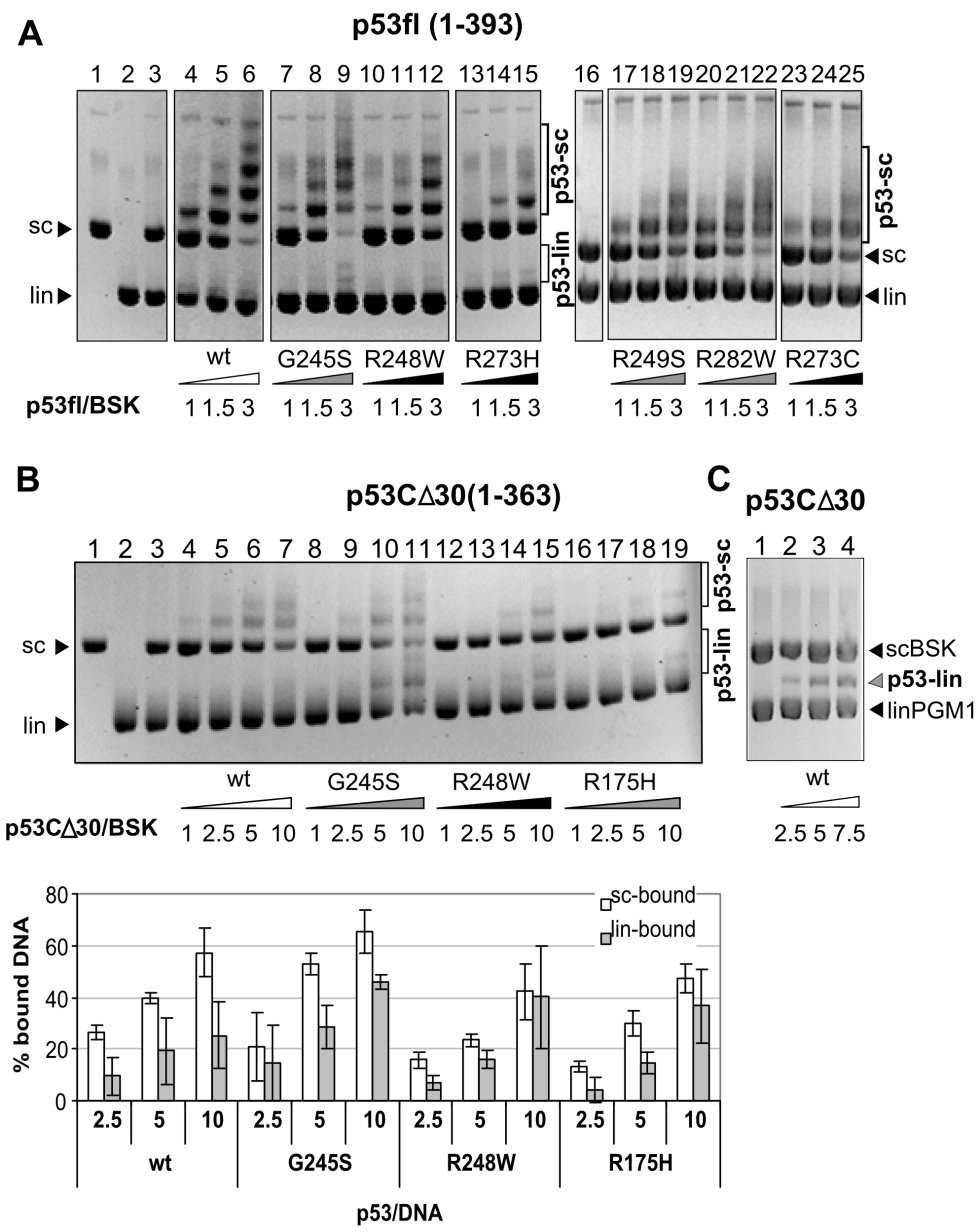
Only full length mutp53 proteins exhibit strong SCS-binding in sc/lin competition assay for scBSK *in vitro*. A) All mutp53fl proteins selectively recognized scBSK by EMSA. P53 proteins (protein amount expressed as p53/total DNA ratio) were incubated with scBSK (200 ng, lanes 1, 3–25) and linBSK (200 ng, lanes 2–25) and separated on a 1.3% 0.33x TBE agarose gel at 4°C (linDNA migrated faster than scDNA). P53-DNA binding was detected by Et-Br staining of DNA. The conformation of p53 proteins is labeled: white (wtp53), grey (conformation mutants) and black (contact mutants). B) Binding of CΔ30-wtp53 (lanes 4–7), CΔ30-G245S (lanes 8–11), CΔ30-R248W (lanes 12–15) and CΔ30-R175H (lanes 16–19) to pBSK in sc/lin competition asssay was performed similarly to p53fl (A). CΔ30-R248W and CΔ30-R175H lose the ability for strong SCS-binding. Column graph below was plotted on the basis of Et-Br stained DNA on agarose gels (from three independent experiments), free DNA substrates labeled with arrows were measured by densitometry and % of bound DNAs (sc and lin) were calculated the same way as in Fig. 2. C) Preferential binding of CΔ30-wtp53 (lanes 2–4) to linPGM1 in sc/lin competition assay with scBSK, complex of p53-linDNA is bonded.

**Figure 4:**
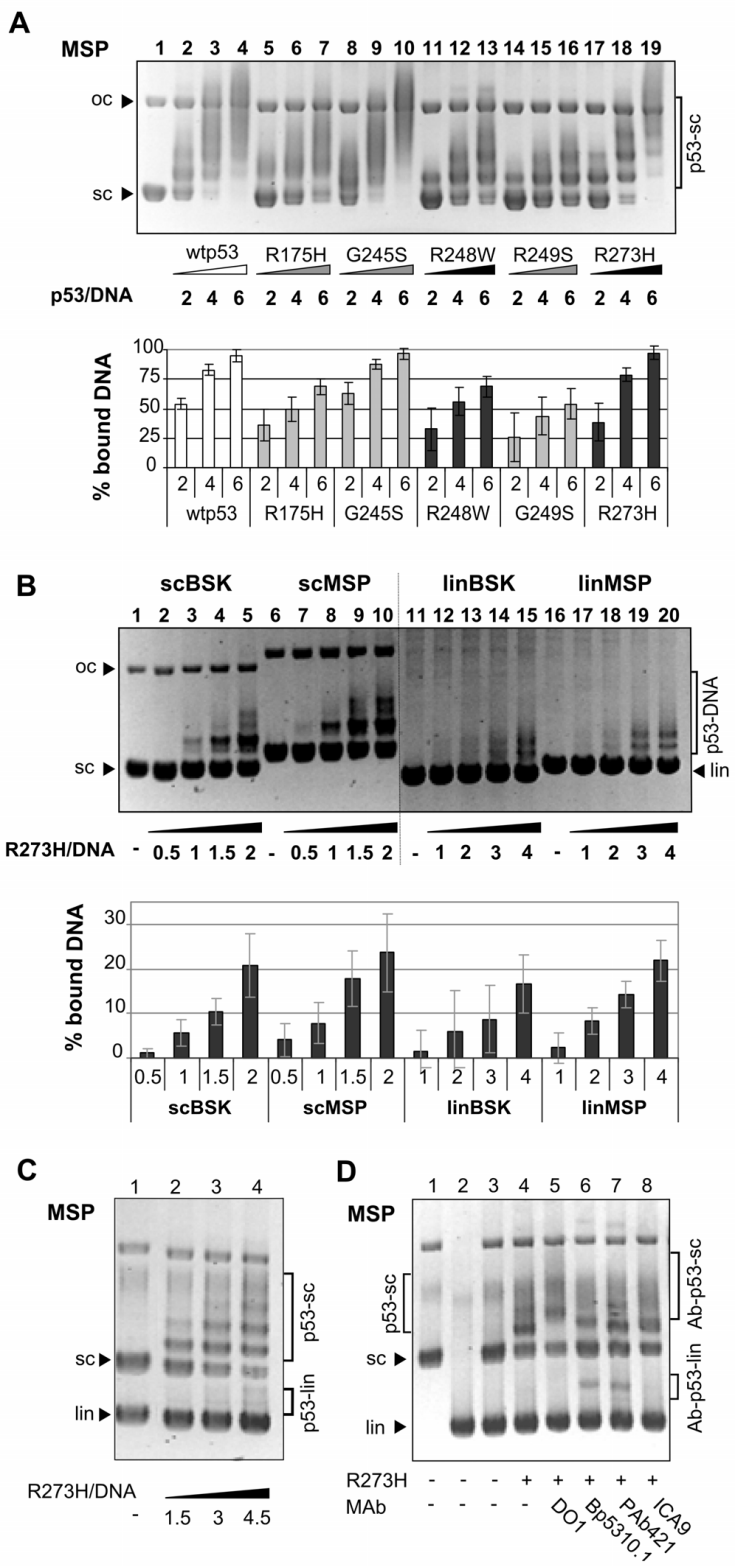
Mutant p53 recognition of MSP/MST1 sites in scDNA. A) Mutp53 proteins recognize MSP/MST1 site in scDNA differently. Mutp53s and wtp53 were bound to scMSP at a molar ratio p53/DNA 2, 4 and 6. B) R273H binding to scBSK (lanes 2–5), scMSP (lanes 7–10), linBSK (lanes 12–15) and linMSP (lanes 17–20) was compared at p53/DNA molar ratios of 0.5–4. P53-DNA binding and EMSA condition for A) and B) were the same as in Fig. 1B. Both graphs show the evaluation of p53-DNA binding (from three independent experiments) as the dependence of % of bound DNA (axis *y*) on the amount of input of p53 proteins in the reaction (expressed by molar ratio p53/DNA, axis *x*). C) Mutp53 R273H binds selectively to scMSP. In competition experiment R273H (lanes 2–4) were bound to a mixture of scMSP and linMSP, experimental condition was the same as in 3A). Observed p53-scDNA complexes and p53-linDNA complexes are marked. Evaluation of binding is shown on Fig. S4C. D) Influence of N- and C-terminal antibodies on SCS-binding of R273H to a DNA mixture of scMSP and linMSP. DO1, Bp5310.1, PAb421 and ICA9 (50 ng, lanes 5–8) were preincubated with R273H (100 ng) at 25°C, sc/lin competition EMSA experiment was performed at RT. Complexes of Ab-p53-linDNA were observed mainly with Bp5310.1 and PAb421 (lanes 6 and 7); control DNAs (sc, lin, sc+lin; lanes 1, 2, 3).

**Figure 5:**
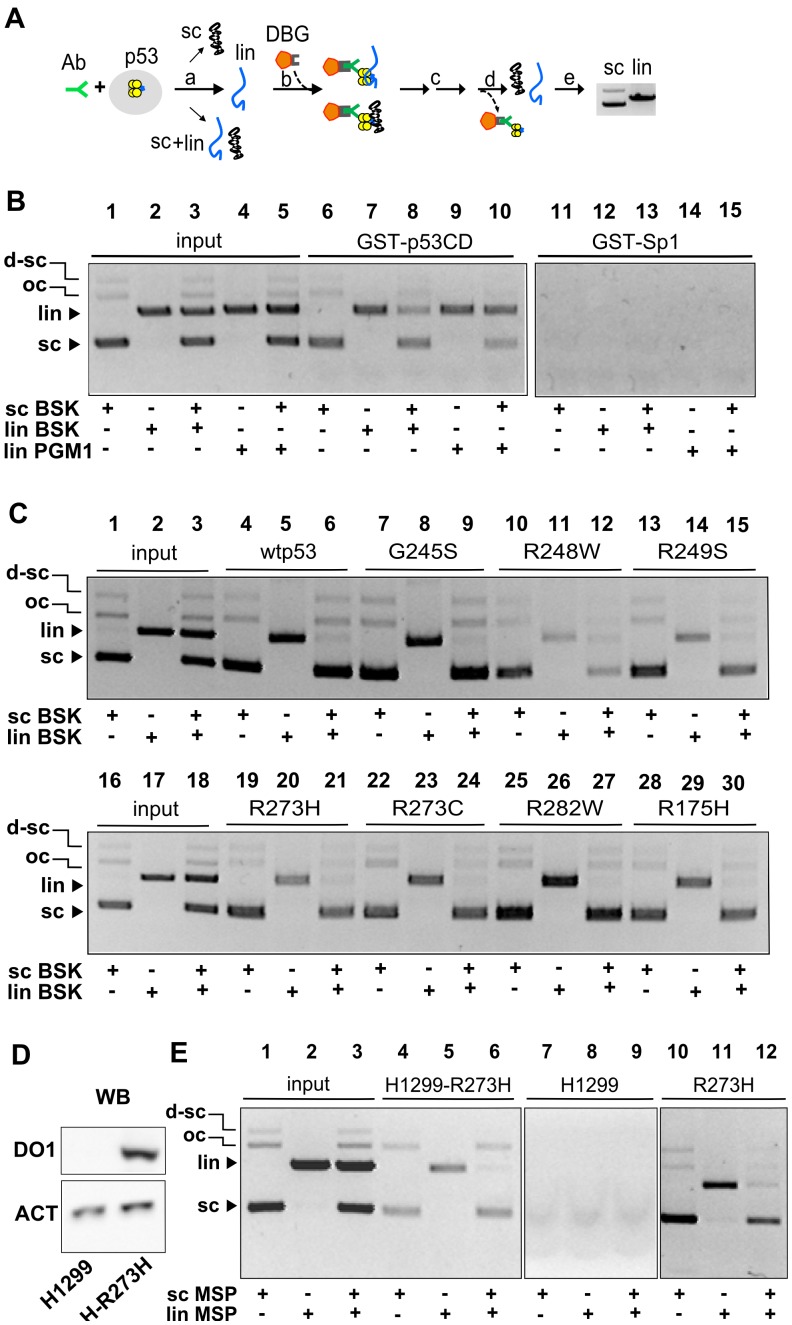
SCS-binding of p53 proteins by magnetic beads-based immunoprecipitation assay (MBIP) *in vitro and ex vivo*. A) Scheme of the procedure (from left to right): the cell lysate or purified proteins were mixed with antibody (Ab) and then with a DNA substrate and incubated to allow formation of the Ab-p53-DNA complex (a). Then, the complex is captured at DBG (magnetic beads-coated with protein G) (b). The bead suspension is washed (c), followed by dissociation of DNA from the complex with p53 (d) and DNA eluted from beads is detected by agarose electrophoresis (e, Et-Br staining). B) Control of specificity of MBIP assay for sc and lin DNAs. Binding of 100 ng of purified GST-p53CD protein to scBSK (lane 6), linBSK (lane 7), linPGM1 (lane 9) and mixtures of scBSK/linBSK (lane 8) and scBSK/linPGM1 (lane 10) by MBIP assay with anti-GST Ab are shown on the left. Lanes 1–5 (scBSK, linBSK, linPGM1 and sc+lin) were a control for input DNA (50 ng, 1/6 input DNA). After anti-GST immunoprecipitation, bound DNAs (lanes 6–15) were analyzed on 1% TAE gel (scDNA migrates faster than lin or oc forms). Binding of GST-Sp1 (construct without DNA binding domain) to different types of DNA (lanes 11–15) by MBIP assay with the same Ab was not observed (right panel). C) Binding of seven purified hot spots and wtp53 to scBSK, linBSK and a scBSK/linBSK mixture by MBIP assay with DO1. Lanes 1–3 and 16–18 are a control for input DNAs (50 ng). D) Control of p53 and actin levels in cell lysates from H1299 and H1299-R273H (induced by tetracyclin for 24 h) by WB with DO1 and anti-Actin antibody. E) Binding of R273H from H1299-R273H lysate to scMSP (lane 4), linMSP (lane 5) and a scMSP/linMSP mixture (lane 6) by MBIP assays. Lanes 1–3 are a control for input DNA (50 ng). Lysate from H1299 cells was used for control reaction for DO1 immunoprecipitation of sc, lin and scMSP/linMSP mixture (lanes 7–9). MBIP assay was performed also with recombinant purified protein R273H (100 ng) with scMSP (lane 10), linMSP (lane 11) or scMSP/linMSP mixture (lane 12). After DO1 immunoprecipitation, DNAs were analyzed on 1% 1x TAE gel (scDNA migrates faster than linDNA, ocDNA or d-sc (dimer of scDNA)).

We investigated the SCS-binding of mutp53 proteins using scBSK/linBSK competition assays, previously used for wtp53 [37], [40], [41]. The Et-Br stained agarose gels (Fig. 3A) show a highly selective scDNA binding of all mutp53 proteins examined at protein/total DNA ratios ranging from 1 to 3, i.e. G245S (lanes 7–9), R248W (lanes 10–12), R273H (lanes 13–15), R249S (lanes 17–19), R282W (lanes 20–22), R273C (lanes 23–25), R175H (not shown) and wtp53 (lanes 4–6). The addition of competitor linDNA did not significantly affect binding of the mutp53 proteins to scDNA, and binding of these proteins to linDNA in the presence of scDNA was negligible (only for G245S, R248W and R273H, faint bands of p53-linDNA complexes were observed at a p53/DNA ratio of around 3, Fig. 3A). The same results were obtained upon competition of scDNA (σ∼−0.05) with relaxed circular duplex DNA (σ = 0) (tested for R273H, G245S and wtp53, not shown). We observed that the preference of mutp53 for scDNA in sc/lin competition experiment was independent of the expression system for the recombinant mutp53 proteins. SCS-binding of mutp53 isolated from insect cells (mutp53i, G245Si [16]) after recombinant baculovirus infection (G245Si, Fig. S2D) was similar to recombinant mutp53 expressed in *E.coli* used for the majority of experiments (Fig. 1, 2, 3, 4, 5). Taken together, SCS-binding is a common attribute of seven hot spot mutp53 proteins *in vitro*.

### Supercoil-selective Binding of Mutant p53 Proteins Requires the CTDBS

To analyze the contribution of the CTDBS of mutp53 to SCS-binding, we used the C-terminally truncated forms of mutp53 R175H, G245S, R248W and R273H (CΔ30-mutp53, aa 1–363, lacking the CTDBS) (Fig. 3B, S3). Under sc/lin competition assay conditions, CΔ30-wp53 (Fig. 3B, lanes 4–7) exhibited reproducible preference for scDNA, as indicated by a higher number and stronger intensity of the retarded bands resulting from p53-scDNA complexes, compared to p53-linDNA (densitometric tracing of the gel revealed about two-times more efficient binding of these proteins to scDNA, compared to linDNA, at molar ratios p53/DNA = 1–10). In contrast, CΔ30-R175H (lanes 16–19), CΔ30-R248W (lanes 12–15), CΔ30-R273H (Fig. S3) bound to both sc and linDNA about equally. At least two-times higher amounts of the CΔ30-mutp53 proteins, compared to the amount of CΔ30-wtp53, were required to obtain a comparable retardation (Fig. 3B and S3A, B). In the case of CΔ30-G245S (Fig. 3B, lanes 8–11) we observed the preference for scDNA at lower p53/DNA ratios (Fig. 3B, lanes 9–10, graph).

This result indicates that in the absence of the CTDBS hot spot mutp53 proteins R175H, R273H and R248W have lost the ability to selectively recognize scDNA.

### Recognition of mutp53 Binding Sites by Hot Spot mutp53 Proteins in vitro

Several mutp53 binding sites were identified by p53 specific chromatin imunoprecipitation in different cell systems for some of mutp53 target genes as shown for some representative examples in Table S1 (MSP/MST1 [28], Id2 [30], PPARGC1A and FRMD5 [19]).

To examine the role of DNA topology in mutp53 recognition of different mutp53BSs we analyzed the interaction of mutp53 proteins with sc and lin forms of mutp53BSs. We observed differences in the recognition of the sc form of mutp53BS-MSP (pMSP; 161 bp fragment of the MSP/MST1 promoter in pBSK [28]) between individual mutp53 proteins: R273H and G245S (and also wtp53) were the strongest scMSP binders (Fig. 4A, lanes 17–19, 8–10, 2–4). We next compared R273H binding to pMSP and pBSK using molar ratios p53/DNA varying from 0.5 to 4. R273H bound scMSP with slightly higher affinity than scBSK (Fig. 4B, lanes 2–3 versus 7–8). Since binding of R273H to linBSK and linMSP was similar (lanes 14–15, 19–20), we conclude that DNA superhelicity facilitated mutp53 R273H binding to the MSP/MST1 site. Finally, sc/lin competition assays with the MSP/MST1 mutp53BS also showed a preference of purified R273H at molar ratio 1.5–4.5 for scMSP (Fig. 4C, lanes 2–4 and Fig. S4C).

Next we used antibodies mapping the epitopes at the p53 C-terminus to evaluate the contribution of CTDBD of R273H to SCS-binding (Fig. S4 and 4D) as was done for wtp53 [41]. Increasing amount of Bp5310.1, PAb421 and ICA9 slightly reduce R273H binding to scDNA (Fig. S4A). In the sc/lin competition assay, Bp5310.1- and PAb421- (mapping to the CTDBS) mediated decrease of binding to scDNA was partially compensated by binding to linDNA. Formation of p53-linDNA bands were observed (Ab-p53-lin; Fig. 4D, lanes 6 and 7; Fig. S4B, lanes 5–8 and 10–12). On the contrary, ICA9 did not produce retarded bands with linDNA (Fig. 4D, lane 8; Fig. S4B, lanes 13–16). N-terminal DO1 mAb strongly affect mobility of p53-scDNA complexes, but do not influence the preference for scDNA (Fig. 4D, lane 5).

Recently, we detected the interaction of mutp53 R273H with repetitive intronic/intergenic DNA as potential mutp53BSs isolated by ChIP from U251 glioblastoma cells (e.g. AA3, AB10, AA12, AA20, Tab. S1) and showed that sc forms of repetitive intronic/intergenic mutp53BSs were better substrates for R273H than the pCRII vector alone [19]. The analysis of R273H binding to scAB10 (a repetitive region from PPARGC1A promoter) and scAA3 are in agreement with our former results (Fig. S5A). Similarly as with the MSP/MST1 site, we observed differences in the recognition of sc forms of mutp53BS (pAA3 and pAA12) between individual p53 proteins: R273H (and also wtp53) were the strongest binders (Fig. S5B) in contrast to R175H.

Thus, recognition of mutp53 binding sites by hot spot mutp53 proteins *in vitro* is positively influenced by DNA superhelicity. C-terminal modifications by PAb421 and Bp5310.1 negatively influence R273H SCS-binding.

### Supercoil-selective DNA Binding of mutp53 Proteins by Immunoprecipitation Techniques in vitro and ex vivo

To analyze SCS-binding of mutp53 directly from cancer cells we used the MBIP assay (an immunoprecipitation technique, see Fig. 5A), published recently for the determination of p53-SCS-binding by purified wtp53 [42], [55]. Selectivity of the MBIP assay was tested with GST-p53CD (aa 92–312) and GST-Sp1 (a construct lacking the DNA binding domain was used as a negative control; Fig. 5B, lanes 11–15). In agreement with our EMSA experiments (CΔ30-wtp53, Fig. 3B, lanes 4–7), p53 containing only core domain slightly preferred the scBSK form in competition with linBSK (GST-p53CD, Fig. 5B, lane 8). Preference for linPGM1 was observed in competition assays with scBSK by both EMSA (CΔ30-wtp53, Fig. 3C, lanes 2–4) and MBIP assay (GST-p53CD, Fig. 5B, lane 10), both CΔ30-wtp53 and GST-p53CD contains aa 92–312 of core domain. MBIP assay with purified proteins confirmed our EMSA data (Fig. 3A) that supercoil-selective binding is a common property of all seven hot spots mutp53s in the sc/lin competition assay (Fig. 5C). Then the MBIP technique was used to study sc, lin and competitive sc/lin DNA binding of mutp53 R273H in cell lysates (R273H stably transfected into human tumour p53-null H1299 cells; Fig. 5D, E) or endogenous R273H, R273C and G245S (glioblastoma cell lines Onda10, U251 and Onda11; not shown). Mutp53 proteins in cell lysates efficiently bound both, the sc and the lin forms of AB10 and MSP/MST1 mutp53BSs (AB10, Fig. S5C lanes 5–6; pMSP, Fig. 5E, lanes 4–5), in the absence of the other DNA form. But in a sc/lin competition experiment, mutp53 proteins with a high preference bound the sc forms of AB10 (Fig. S5C, lane 7) and pMSP (Fig. 5E, lane 6).

Hence, sc form of mutp53BSs in competition experiment were bound by mutp53 proteins expressed in human cancer cells with a high preference similarly as observed for the purified p53 proteins.

### Supercoil-selective DNA Binding of mutp53 R273H and G245S in H1299 Cells

**Figure 6:**
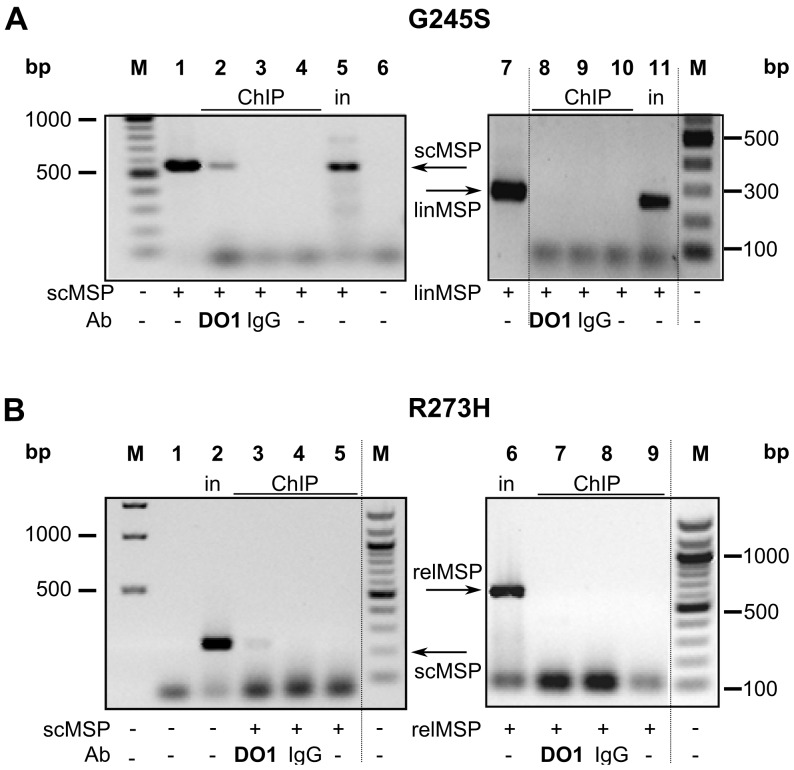
Influence of DNA topology on mutp53 MSP/MST1 recognition in H1299 cells. A) Preferential binding of G245S to scMSP in sc/lin competition assay in H1299 cells by ChIP. pGL3-MSP (sc, 2 µg), pMSP (lin, 2 µg) and pCDNA3.1-G245S (2 µg) were co-transfected into H1299 cells. ChIP assay was performed after 20 h. DNAs (sc or lin) bound by mutp53 were detected by two (sc and lin) specific PCRs on 1.5% agarose gels. Left side shows detection of scDNA by PCR with GL2 and RV3 primers: control DNA for transfection (lane 1, scpGL3-MSP); 1/20 of ChIP input DNA (lane 5); as ChIP were marked all immunoprecipitation samples: DO1-Ab (lane 2, bound scDNA), whole mouse IgG-Ab (lane 3, negative control), IP without Ab (lane 4, negative control); negative control of PCR (lane 6). Right side shows detection of linDNA by PCR with BT3 and MSP primers: control DNA for transfection (lane 7, pMSP/SmaI); 1/20 of ChIP input DNA (lane 11); as ChIP were marked all immunoprecipitation samples: DO1-Ab (lane 8, bound linDNA), whole mouse IgG-Ab (lane 9), IP without Ab (lane 10). Results of PCR analysis of immunoprecipited DNA were detected on a 1.5% agarose gel. Samples for PCR on the gel are: plasmids (lane 1 (scpGL3MSP), lane 7 (linMSP)); 1/20 of input DNA (lanes 5, 11 marked as in); IP without Ab (lanes 4, 10); IP from DO1-Ab (lanes 2, 8); IP from whole mouse IgG-Ab (lanes 3, 9). (B) Preferential binding of R273H to scMSP in sc/rel competition assay in H1299 cells by ChIP. pMSP (sc, 2 µg), pGL3-MSP (rel, 2 µg) and pCDNA3.1-R273H (2 µg) were co-transfected into H1299 cells. Other condition was the same as in A). DNAs (sc or rel) bound by mutp53 were detected by two (sc and rel) specific PCRs. Left side shows detection of scDNA by PCR with BT7 and MSP primers: 1/20 of ChIP input DNA (lane 2); as ChIP were marked all immunoprecipitation samples: DO1-Ab (lane 3, bound scDNA), whole mouse IgG-Ab (lane 4), IP without Ab (lane 5); negative control of PCR (lane 1). Right side shows detection of relDNA by PCR with GL2 and RV3 primers: 1/20 of ChIP input DNA (lane 6); as ChIP were marked all immunoprecipitation samples: DO1-Ab (lane 7, bound relDNA), whole mouse IgG-Ab (lane 8), IP without Ab (lane 9).

To confirm supercoil-selective binding of mutp53 proteins in cells, we cotransfected an equimolar mixture of sc and lin/rel forms of the MSP/MST1 mutp53BS in different vector backgrounds (pGL3 and pBSK) into H1299 cells together with mutp53 expression vectors (pCDNA3.1-G245S or R273H). Binding of G245S to scpGL3-MSP/linMSP and R273H to scMSP/relpGL3-MSP was studied by ChIP assay performed after 24 h with DO1 and control antibody (whole mouse IgG). Significant preference for the sc form of MSP/MST1 site was observed for both G245S (Fig. 6A, lane 2) and R273H (Fig. 6B, lane 3). Different specific primers were used for sc forms of the MSP/MST1 site in pGL3 (Fig. 6A) and pBSK (Fig. 6B) vectors. Binding to linMSP (Fig. 6A, lane 8) or relMSP (Fig. 6B, lane 7) was not detected. The PCR signals obtained from mouse IgG antibody precipitation and the negative control did not exceed background level. The preference of mutp53 for the sc form of the MSP/MST1 mutp53BS was unchanged by vector background (Fig. 6A, scMSP in pGL3 vector; Fig. 6B, scMSP in pBSK).

### Impact of BAX and MSP/MST1 Promoter Topology on mutp53-driven Gene Repression

To analyze, whether the DNA topology of a p53 binding site (p53BS) has an effect on (mut and wt) p53-driven transcription, luciferase reporter assays were performed using supercoiled, relaxed or linearized reporter vectors, pGL3-BAX, pGL3-MDM2 and pGL3-MSP. Equal amounts of the described reporter vectors were transfected, either in their sc, lin (not shown) or rel forms, together with plasmid pRLSV40 (internal control) and p53 expression plasmids. At first, we investigated the effect of DNA topology on mutp53 repression of the BAX promoter region, as described for hot spot mutp53 proteins by [56]. Transfected H1299 cells were harvested 16–20 h after transfection; during this short term incubation the scDNA was not progressively relaxed and shows an intermediate level of nucleosomal assembly, as shown previously [57], [58], [59].

**Figure 7:**
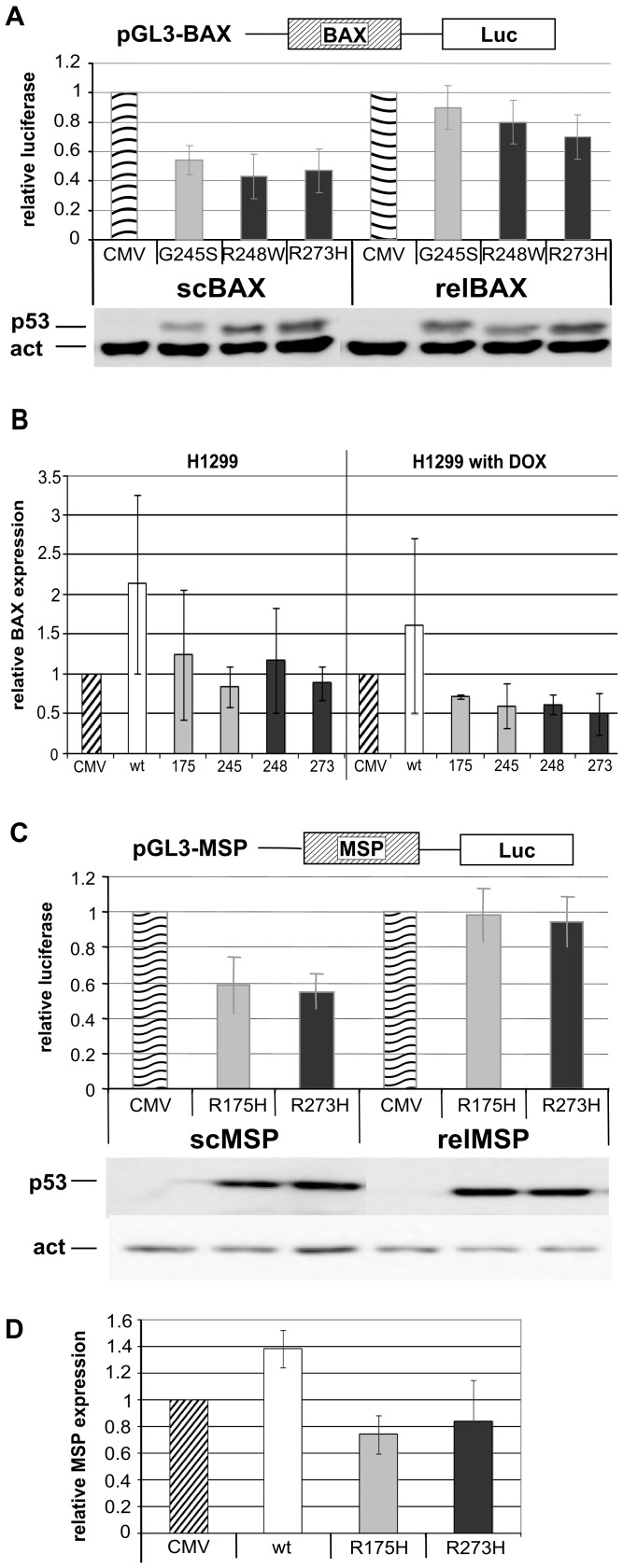
Influence of DNA topology on mutp53-driven repression of BAX and MSP/MST1 promoters and mutp53 mediated down-regulation of *BAX* and *MSP/MST1* expression. A, C) Influence of DNA topology on mutp53-driven repression of BAX (A) and MSP/MST1 (C) promoters. Saos2 or H1299 cells were transiently transfected with plasmids expressing the p53 constructs (based on pCDNA3.1) or pCDNA3.1 vector alone (CMV) together with the reporter plasmids expressing the firefly luciferase gene under the transcriptional control of the indicated gene promoters (BAX, MSP/MST1) and a reference plasmid with the renilla gene under control of the SV40 promoter. Luciferase activity was analyzed 16–20 h after transfection as described in Material and methods. Transfections were carried out in triplicates and at least three independent times and standards deviations are indicated. Representative western blot analysis was performed using 50 µg of samples from the transfection to determine the expression status of p53. A) The BAX promoter in the pGL3-basic vector was repressed by mutp53 (R248W, G245S and R273H) more efficiently in scDNA form (left side) in comparison with relDNA form (right side) in H1299 cells. C) MSP/MST1 promoter (161 bp mutp53BS in pGL3-promoter vector) was transfected to Saos2 cells in sc (left side) or relaxed (right side) forms. Mutp53 proteins (R175H and R273H) repress MSP/MST1 promoter in scDNA form more efficiently than rel form in Saos2 cells. B, D) Analysis of down-regulation of BAX and MSP/MST1 mRNA levels in cells overexpressing mutant p53s. H1299 (B) and Saos2 (D) cells were transfected by p53 constructs or empty vector in the same conditions as A) and C). Samples marked DOX were additionally exposed 0.1 µM doxorubicin for 16 h. Total RNA was isolated, and mRNA levels of BAX (B) and MSP/MST1 (D) were determined by quantitative real-time reverse transcription PCR. BAX and MSP/MST1 values were normalized by GAPDH, HPRT1 or Actin. The values are the average of three biological independent experiments.

Expression of mutp53 (G245S, R248W and R273H) resulted in the repression of the sc form of the pGL3-BAX reporter, compared to vector and in accordance with findings obtained earlier [56]. Repression of the relaxed form of the pGL3-BAX reporter by mutp53 proteins was significantly weaker (Fig. 7A). Firefly luciferase activity of pGL3 vectors was normalized to renilla luciferase activity and transfection efficiency of sc, lin and rel plasmids was analyzed by PCR of isolated DNA from cell lysates (for sc and rel forms of pGL3-BAX Fig. S6A). In the case of MDM2 promoter, repression by R273H and R248W was very weak, in contrast to residual activation by G245S and strong activation by wtp53 (Fig. S6B). In agreement with the original observation, wtp53 transactivated better the relaxed form of the promoter [60].

Finally, sc and rel forms of the MSP/MST1 binding site in the pGL3 promoter vector were transfected into Saos2 cells and luciferase assays were performed under the same condition as described above for pGL3-BAX. The strongest repression of the pGL3-MSP reporter was observed for its sc form upon R175H and R273H expression (Fig. 7C, left part) compared to the rel form of pMSP (Fig. 7C, right part) or pGL3-basic and pGL3-promoter alone (Fig. S6C).

To confirm the influence of p53 mutants on transcriptional activity of BAX and MSP/MST1 in the studied systems, we analyzed the effect of exogenous mutp53s and wtp53 on expression levels of endogenous target genes in H1299 and Saos2 using quantitative RT-PCR (qRT-PCR). Cells were transfected with vectors bearing coding sequences for wtp53, particular mutp53s or a pCDNA empty vector in conditions similar to the luciferase reporter assays. Alterations in *BAX* [54] and *MSP/MST1* gene expression, induced by wtp53 and mutp53, were compared against the level of mock transfection (pCDNA empty vector); both unstressed (Fig. 7B, D) and doxorubicin treated cells (Fig. 7B and S7A, B) were measured. We observed mutp53-dependent down-regulation of *MSP/MST1* in unstressed Saos2 and in H1299 cells (Fig. 7D and S7B). The level of MSP/MST1 repression differed between mutp53s and stressed/unstressed conditions. The most consistent repression was observed with doxorubicin-stressed cells (Fig. S7B) and with both G245S and R175H in all experimental systems (Fig. 7D, S7B). Down-regulation of *BAX* was detected mainly in doxorubicin-stressed cells (Fig. 7B and S7A). Generally, the results obtained by qRT-PCR (Fig. 7B, D, S7) at least partially correlated with the results of luciferase assay (Fig. 7A, C). *MSP/MST1* and *BAX* were down-regulated by mutp53s under a subset of conditions.

We have shown that DNA topology influences the mutp53-driven repression on the system of BAX and MSP/MST1 promoters, regulated by mutp53s in H1299 and Saos2 cells.

## Discussion

In our previous studies we described the selective binding of the tumor suppressor p53 to topologically constrained DNA [19], [39]. Other authors reported that the DNA topology not only influences the selectivity of wtp53-DNA binding but also wtp53 driven transactivation [60]. So far, no systematic study has focused on hot spot mutp53 recognition of topologically distinct DNA molecules in contrast to the intensive investigations on wtp53 and mutp53 sequence-specific and/or structure-selective DNA binding (reviewed in [26], [27]). Mutp53 transcriptional regulation of target genes has been established as an important attribute of mutp53s (reviewed in [24], [25], [27], [61]). Recently, many studies concentrated on the investigation of co-operative interactions of mutp53 with sequence-specific transcription factors on DNA. However, a universal mechanism that would operate across different experimental systems is still missing. Direct interaction of mutp53 with structure-specific DNA or direct recruitment of mutp53 to unique sequence-specific elements and/or unique chromatin landscapes all belong to additionally proposed mechanisms. It is still thought that specificity of mutp53 for certain regulatory sequences is mediated through preferential binding to non-B DNA structural motifs rather than to specific consensus sequences [27]. Formation of the majority of non-B DNA structures is promoted by DNA negative superhelicity [33] and may be physiologically important and pathologically significant [35]. The genomic DNA in eukaryotic cells is organized in supercoiled chromatin fibers, which undergo dynamic changes during DNA metabolic processes such as transcription or replication [35].

Considering these facts, we investigated the ability of hot spot mutp53 proteins to bind selectively scDNA. Furthermore, we studied the influence of DNA topology on mutp53BS recognition both *in vitro* and in cells.

### Recognition of Topologically Constrained DNA by mutp53

Here we show for the first time that hot spot mutp53 proteins (R175H, R248W, G245S, R249S, R273H, R273C and R282W) are inherently capable of structure-selective binding to supercoiled plasmid DNA independently of the sequence context by EMSA and MBIP methods. This binding varies somewhat depending on the p53 mutation, but changes dramatically with the topological state (superhelix density) of the DNA. We observed slight differences among the p53 mutants in scDNA binding, without any apparent correlation with the type of mutation (DNA contact or conformation, see Fig. 2). Only the R175H protein bound all DNA forms with lower apparent affinity than the other mutp53 proteins. So far, only an identical binding pattern of mutp53 R249S and wtp53 to scDNA was observed early by [36]. Some of the mutants retained certain ability for recognizing p53CON in longer DNA (∼3kbp; not shown for 50 bp, 500 bp). Among the mutp53 proteins, mutp53 G245S and R273H exhibited the strongest binding to p53CON in longer DNA. This is in good agreement with previous results with p53CON within DNA molecules longer than 35 bp [15], [62].

In our previous work [37], [40] we identified two distinct types of wtp53 preferential binding to scDNA: first, highly preferential (supercoil-selective) scDNA binding by proteins retaining the ability of oligomerization and possessing active CTDBS (even those lacking the core domain) and second, slightly preferential scDNA binding through the p53 core domain in the absence of the CTDBS (e.g. CΔ30p53 and GST-p53CD). From this point of view, it is not surprising that all full length mutp53 proteins in this study bound to scDNA with a high selectivity, as did wtp53 [37], [40]. On the contrary, the C-terminally truncated mutants (CΔ30-R175H, CΔ30-G245S, CΔ30-R248W and CΔ30-R273H) differed in their level of preference for scDNA in sc/lin competition experiment (Fig. 3B). Most of them did not exhibit preference for scDNA, with the exception of CΔ30-G245S. Moreover, it was shown that the “wild-type” function of G245S was easily restorable by small molecules [11], [63]. In addition, G245S efficiently recognized non-B DNA (four-way junction and stem-loop structures [16]) and differed from other mutants by its strong ability to form higher oligomeric forms of mutp53-DNA complexes [16].

### Mutant p53 Binding Sites, DNA Supercoiling and non-B DNA Structures in mutp53-DNA Interaction

Using chromatin immunoprecipitation technique and chip-on-chip analysis increasing numbers of mutp53 binding DNA regions (mutp53BS) were identified in cells (reviewed in [24], examples Tab. S1). Direct non-B DNA structure-specific binding of mutp53 was described in details for four-way junctions, stem-loop structures [16], G-quadruplexes [17] and repetitive genomic sequences: MARs [20] and other DNA elements isolated by ChIP [19], [22], all with a high propensity to form non-B DNA structures (e.g. DNA cruciforms, DNA triplexes or DNA quadruplexes) under superhelical stress. Previously, we have shown, that R273H binding to a small set of repetitive ChIP sequences (AB7, AA3, AA12 and AB23) in vector pCRII was stronger than to pCRII alone [19]. Wtp53, R249S and R273H bound to two sequences (pCRII-AA3 and pCRII-AB23) in linearized form with comparable affinity, whereas R175H bound very weakly. Similarly to these findings with repetitive elements [19] and to previously published data with different p53CON sequences [15], we observed the greatest differences in the ability of individual mutp53 proteins to recognize in scDNA the putative mutp53 binding site from MSP/MST1 promoter. The strongest binders were R273H and G245S, while R175H, R248W and R249S bound the same DNA target with lower, but mutually comparable affinities. C-terminal modification of R273H negatively influences preference for scDNA containing MSP/MST1 sequence, but its residual interaction suggests involvement of the core domain in scDNA recognition as was observed for MAR sequences [20].

Altogether, these data suggest that mutp53-DNA binding is determined by combined effects of topological features of the DNA and presence of certain DNA sequences [16], [18], [19], [20], [22], [27]. Computational analysis (MAR-WIZ [64], SIDD [65], non-B DNA Motif Search Tool [66], triplex search tool [67]) of potential mutp53BSs (Fig. S8 and Tab. S1) from several mutp53 target genes confirmed our hypothesis that mutp53BSs has potential to form non-B DNA under superhelical stress. Predicted supercoil-stabilised non-B DNA potential of MSP/MST1 and AA3 sequences was detected also by S1 nuclease treatment of plasmid DNA (Fig. S8C). Also short repetitive palindromic regions in commonly used plasmid DNAs (e.g. pBSK) in supercoiled form can create a non-B DNA conformation, as we show in Figure S8B, C (detected by computational prediction and S1 nuclease treatment). In addition to the supercoil-stabilized local non-B structures, several global structural features in the supercoiled DNA were proposed to be recognized by p53 proteins [39], [40], [68], including segments involving two closely positioned duplexes, bent DNA at the superhelix apices, DNA crossovers (occurring in both negatively and positively scDNA [36], [39], [68]) and open DNA structures such as cruciforms.

### Transcriptional Regulation by Mutant p53

Mutp53 proteins exert gain-of-function also by positive/negative modulation of gene expression [23], [24], [25], [61], [69], [70], [71], [72], [73]. Previously, it was shown that the anti-apoptotic gain-of-function activity of mutp53 is due, at least in part, to repression of *CD95/Fas/Apo1*, *BAX* and *MSP/MST1* gene transcription by mutp53 [28], [29], [56], although the mechanism of this repression is still not clear. For the CD95/Fas/Apo1 and BAX promoters it was suggested that mutp53 repression might be mediated, at least in part, through interaction with p53 family members (TA forms of p63 and p73) [74]. Another possible mechanism could be the direct DNA binding of mutp53 to regulatory regions of the repressed genes [24]. No information on such mutp53 specific sequences in e.g. the BAX promoter has been reported so far, but recently a functional MAR element was located in the BAX promoter region [75]. It seems that mutp53 and wtp53 binding sites can be separated, as was recently shown for the *CD95/Fas/Apo1* gene [74]. Our study of the influence of DNA topology on gene repression was based on the described association of mutp53 (R175H and R273H) with a 161 bp long part of the MSP/MST1 promoter in cells [28]. We used an experimental system in which mutp53 expression down-regulated endogenous *MSP/MST1* genes (p53 null-cells: H1299 and Saos2). Our luciferase assay data show that the strongest mutp53 protein R273H repression was for the sc form of the reporter containing the mutp53-MSP binding site. These data correlated with *in vitro* and in cells binding studies. R273H, the best binder to MSP/MST1 binding site in scDNA, is also a good repressor of the MSP/MST1 reporter. Furthermore, in *ex vivo* binding assays, R273H from several cancer cell lines displayed significant SCS-binding also in an in cells setting. In case of the BAX promotor, transrepression by hot spot mutp53s has been observed by different laboratories using the luciferase assay [56], [74], [76]. Regulation of endogenous *BAX* is more complicated *in vivo*, where many other transcription factors are involved (e.g. SP1, E2F1, HMGB1 [77], [78], [79]). In our case, we observed marginal repression of endogenous *BAX* by mutp53 in unstressed conditions; repression effect was strongly increased after application of doxorubicin-treatment.

Our transrepression study of BAX promotor by G245S, R248W and R273H corresponds with previous research performed also with sc forms of reporters [56], [74], [76]. Also some genomic repetitive fragments were repressed by mutp53s (AA12, Fig. S6C [19]). These data are in good agreement with our previous study which showed that mutp53 (R273H) interaction with structurally flexible repetitive genomic DNA elements (ChIP genomic DNA fragments) in the nucleus of U251 glioblastoma cells participates in the regulation of gene transcription [19]. Mutp53 R273H was bound to the nuclear scaffold and components of the transcription machinery, including Sp1, YY1 and RNA polymerase II, causing mainly repression of newly identified target genes (e.g. *PPARGC1A* and *FRMD5*). Interestingly, *in vitro* mutp53 R273H interaction with ChIP-sequences, intronic regions of regulated genes, is positively influenced by the presentation of the genomic elements in supercoiled form to a significant extent [19]. We suppose that both, the mutant core domain and an intact C-terminus, contribute to the recognition of genomic DNA as was previously reported for mutp53 specific binding to nuclear matrix DNA (MARs elements, [21], [80]). The C-terminus of mutp53 proteins is strictly required also for the activation of mutp53 target genes such as *EGFR*, *MDR-1* and *c-myc* (reviewed in [69], [72], [81]). Interestingly, it was shown that a C-terminal deletion of p53 impaired the ability of the R175H and R273H p53 mutant proteins to drive invasion and correlated with their ability to inhibit the transcriptional function of TAp63 [82]. We suppose that the strong binding of the C-terminus to supercoiled DNA also plays an important role in repression of the studied *MSP/MST1* and *BAX* genes. Based on these recent findings we hypothesize that abundant (overexpressed) mutp53 in cells can interact stably *in vivo* with already existing non-B DNA structures formed within chromatin filaments due to DNA supercoiling, specific DNA sequences (e.g. BAX and MSP/MST1 promoters, repetitive DNA, MARs) and combinations of other transcription factors. The bound proteins can physically cover the DNA by forming higher-order oligomeric forms (DNA-protein filaments: wtp53 [19], [68] and mutp53 (M. Brazdova unpublished), thus physically blocking transcription of genes. Coverage of chromatin filaments may also lead to e.g. protection of their association with active transcription factors. Recently, co-aggregation of structurally destabilized p53 mutants with wtp53, p63 and p73 was presented as a novel disease mechanism for mutp53 gain-of-function. DNA contact mutp53 R273H and R248W, good binders of scDNA, were shown as predominantly nuclear and non-aggregating in cells [83]. Moreover, there may be the interplay between mutp53-DNA interactions and factors that directly control the DNA superhelicity level in chromatin. In this regard, it has been shown that R273H and G245S, similarly as wtp53, can interact with human topoisomerase I and HMG proteins [84], [85]. It was also reported that p53 stimulates topoisomerase I by modulating its DNA binding [86]. Enhanced topoisomerase I activity can lead to genetic instability by stimulation of non-homologous recombination and gene amplification [87], [88].

### Conclusions

In summary, we show that the hot spot mutp53 proteins (as full length proteins possessing intact C-termini) retain the ability of wtp53 to bind with high selectivity to scDNA. Moreover, preferential binding to more tightly packed DNA molecules containing mutp53 MSP/MST1 binding site was detected also in cells by chromatin immunoprecipitation techniques. We hypothesize that DNA topology is important for mutp53 recognition of cognate sites and may significantly contribute to repression of mutant p53 target genes.

## Supporting Information

Figure S1 **Purity of p53 and CΔ30-p53 proteins.** Purity of recombinant full length p53 proteins (lanes 1–7) and p53CΔ30 forms (lanes 8–11) in quantities ranging between approximately 750 and 1000 ng, is demonstrated on Coomassie blue stained 12.5% SDS-polyacrylamide gels. Standard polypeptide markers (lanes M) and 1 µg of BSA were used as controls.
(TIF)

Figure S2 **Comparison of mutp53 binding to scDNA and linDNA by EMSA and binding of baculoviral p53 protein G245Si to DNA (scDNA, linDNA, and their mix in sc/lin competition assays).** **A–C)** Mutp53 proteins G245S, R273H and wtp53 were bound to scDNA (pBSK, 200 ng), linDNA (linBSK, 200 ng,) and to linDNA with p53CON sequence (linPGM1, 200 ng) in p53/DNA molar ratios as indicated in the figure for 20 min at 4°C before separation on 1% 0.33x TBE agarose gel. Graphs on the right were plotted on the basis of Et-Br stained agarose gels; free DNA substrates labeled with arrows were measured by densitometry. Graphs show the evaluation of p53-DNA binding as the dependence of % of bound DNA (axis *y*) on the amount of input of p53 proteins in the reaction (expressed by molar ratio p53/DNA, axis *x*). Bound DNA (%) were calculated as % of decrease of free DNA after binding of p53 in comparison to input DNAs (lanes 1,7 or 13 0% of bound DNA). **D)** Binding of mutp53i protein isolated from insect cells infected recombinant baculovirus. G245Si was incubated with 200 ng of scDNA (pBSK, lanes 5, 6), 200 ng linDNA (pBSK/SmaI, lanes 2, 3), both (mixture of 200 ng pBSK and 200 ng pBSK/SmaI, lanes 7–9) at molar ratios of p53/DNA 0.5–2, as indicated in the figure, for 20 min at 4°C before separation on 1.3% 0.33x TBE agarose gel. Binding of G245Si to DNA was detected by ethidium bromide staining (linDNA migrated faster than scDNA).
(TIF)

Figure S3 **Binding of CΔ30-R273H to scDNA, linDNA, and their mix in sc/lin competition assays. A, B)** Effect of CΔ30 deletion on p53 protein binding to scBSK and linBSK (similarly to Fig. 3B). CΔ30-R273H protein was incubated with 200 ng of scDNA (pBSK, A) or 200 ng linDNA (pBSK/SmaI, B) at molar ratios of p53/DNA, as indicated in the figure, for 20 min at 4°C before separation on 1% 0.33x TBE agarose gel. **C)** Loss of SCS-binding of CΔ30-R273H. Binding of CΔ30-R273H to scDNA in the presence of linDNA (sc/lin competition assay, mixture of 200 ng pBSK and 200 ng pBSK/SmaI) at molar ratios of p53/DNA, as indicated bellow, was detected in the 1.3% 0.33x TBE agarose gel by ethidium bromide staining (linDNA migrated faster than scDNA).
(TIF)

Figure S4 **Influence of C-terminal antibodies on binding of R273H to scDNA and sc/lin competition. A)** R273H (100 ng) was pre-incubated with 50–200 ng of Bp5310.1 (lanes 3–6), PAb421 (lanes 8–11) and ICA9 (lanes 13–16) at 25°C; scDNA binding expreriment (200 ng pBSK) was performed at RT for 5 h in 1% 0.33x TBE agarose gel. Controls: DNA (lane 1); complex without Ab (lanes 2, 7 and 12). **B)** Similarly R273H (100 ng) was pre-incubated with 50–300 ng of Bp5310.1 (lanes 4–8), PAb421 (lanes 9–12) and ICA9 (lanes 13–16) at 25°C; sc/lin DNA binding expreriment (200 ng pBSK and 200 ng pBSK/SmaI) was performed at 4°C for 8 h in 1.5% 0.33x TBE agarose gel. Complexes of Ab-p53-linDNA were observed with Bp5310.1 (lanes 5–8) and PAb421 (lanes 10–12). Controls: DNAs (sc, lin and their mixture; lanes 1, 2, 3); p53+sc/lin mixture (lane 4). **C)** Evaluation of binding of R23H to MSP/MST1 mutp53BS in competition assay from Fig. 4C, condition for evaluation same as in Fig. 2, S2.
(TIF)

Figure S5 **Mutant p53 recognition of mutp53BS (repetitive ChIP sequences) in scDNA**. Repetitive ChIP sequences scAA3, scAB10, scAA12 were isolated as R273H binding sites (Tab. S1) from U251 cells [19] and cloned to pCRII vector. **A)** R273H binding to scpCRII (lanes 2–3), scAA3 (lanes 5–7), scAB10 (lanes 9–11) was compared at p53/DNA molar ratios of 5–10. **B)** Mutp53 proteins (R273H and R175H) and wtp53 bound scDNA form of scAA3 and scAA12 differently; binding to linear form was already shown [19]. Mutp53s and wtp53 were bound to scAA3 and AA12 at a molar ratio p53/DNA 3, 6, 9 and 12 and 150 mM KCl. P53-DNA binding and EMSA condition for **A)** and **B)** were the same as in Fig. 1. **C**) Binding of R273H from H1299 lysate to scAB10 (lane 5), linAB10 (lane 6) and to scAB10/linAB10 mixture (lane 7) by MBIP assays. Lanes 2–4 (scAB10, linAB10 and their mixture) were a control for input DNA (50 ng, 1/6 input DNA). After DO1 immunoprecipitation DNAs were analyzed on 1% TAE gel (scDNA migrates faster than lin or oc forms). The same conditions as in Fig. 5.
(PDF)

Figure S6 **Influence of DNA topology on mutp53-driven trans-activation/repression measured by luciferase reporter assay. A)** PCR control of DNA transfection for experiments on Fig. 7A. H1299 cells were transiently transfected with p53 expression plasmids (pCDNA) together with pGL3-BAX reporter plasmids (sc and rel form) and a reference plasmid with the renilla gene under control of the SV40 promoter. PCR analysis of isolated DNA from transfected cells was done with GL2 and RV3 primers. More details for transfection are on Fig. 7A. **B, C)** Dual Luciferase Assay showing influence of p53 proteins on gene promoters (pGL3-MDM2, pGL3-promoter, pGL3-basic, pGL3-AA12). H1299 cells were transiently transfected with p53 expression plasmids based on pCDNA3.1 or pCDNA3.1 alone (CMV) together with the reporter plasmids expressing the firefly luciferase gene under the transcriptional control of the indicated gene promoters and a reference plasmid with the renilla gene under control of the SV40 promoter. Experiments were analyzed 16–20 h post transfection and carried out in triplicates and at least three independent times; standards deviations are indicated. Representative western blot analysis of p53 and actin was performed using 50 µg of samples. **B)** Effect of MDM2 promoter topology on p53 transcriptional regulation. Both wtp53 and G245S activated scMDM2 and relMDM2. But R248W and R273H effects on sc and rel form of pGL3-MDM2 reporter were moderate. **C)** Effects of wtp53 and mutp53 on vectors pGL3-basic, pGL3-promoter and repetitive ChIP sequences pGL3-AA12.
(TIF)

Figure S7 **Levels of down-regulation of BAX and MSP/MST1 mRNA in H1299 and Saos-2 cells overexpressing Mutant p53s without stress and after doxorubicin treatment.** H1299 and Saos2 cells were transfected by p53 constructs or empty vector in the same conditions as in Fig. 7 and exposed to 0.1 µM doxorubicin for 16 h ([54], samples marked DOX). Total RNA was isolated and mRNA levels of BAX (**A**) and MSP/MST1 (**B**) were determined by quantitative real-time reverse transcription-PCR. BAX and MSP/MST1 values were normalized by GAPDH, HPRT1 or Actin. The values are the average of three biological independent experiments.
(TIF)

Figure S8 **Non-B DNA structures in scDNA (pBSK, pPGM1, pMSP and pAA3) predicted by computational methods and detected by S1 treatment. A)** *In silico* analysis of MSP/MST1 (chr3∶49,726,122-49,728,196) [28] and AA3 chip fragment (chr5∶81,887,426-81,889,425) [19] as DNA segments containing mutp53 binding sites (mutp53BS). Presence of putative S/MAR elements, regions with superhelical stress induced DNA destabilization (SIDD) and DNA triplexes (TD) was detected by the available tools: MAR-WIZ [64], a dynamic programming algorithm for identification of triplex-forming sequences [67] and SIDD [65]. The results obtained from these tools are summarized in diagrams showing the positions of mutp53BS (blue box), MAR/SAR (orange box) and regions with high SIDD (pick). **B)** The sequence of pBSK was analyzed using the UNAFold software package [89]. Whole-sequence folding prediction at 37°C yielded regions with predicted base-pairing (forced to a maximal distance of 64 bp). These regions were cut out and the free energy of structures formed by the used sequencesm was estimated using the program hybrid-ss from the UNAFold package. Position of the central base and the free energy of each structure are labeled on the outside of the plasmid and given with the sequences in the inserted table. In parallel, we scanned the sequence with a 48 bp sliding window and calculated the same parameters as above for each of the windows, obtaining a sliding numerical “folding potential” for every position on the plasmid. The value of the folding potential is shown grey-coded on the inside of the plasmid. Computational analysis predicted the presence of three regions with significant potential for hairpin formation, regions close to the origin and positions 1050 and 1750. Plasmids pPGM1 and pPGM2 contained inserts with a 26 bp palindromic sequence with a free energy estimated to be −11.6 and −11.8 kJ/mol, respectively. These values rank 4th compared with other similar sites in the pBSK-derived vectors. **C)** ScDNAs (pPGM1, pPGM2, pMSP, pAA3, pBSK) were treated with S1 nuclease followed *ScaI* restrictase digestion [38], [43]. Detection of two fragments (1126 and 1835 bp) indicates DNA cruciform formation in the p53CON site in the case of pPGM2 (lane 5) or AA3 (lane 12). But also pPGM1 (lane 2), pMSP (lane 8) and pBSK (lane 16) were sensitive to S1 nuclease treatment; two pairs of fragments (black arrows) were detected, indicating that pBSK bases can form some non-B DNA structures with unpaired bases, linBSK is 2961 bp long.
(PDF)

Table S1 **Examples of mutant p53 binding sites identified by ChIP and confirmed by** ***in vitro*** **mup53 binding analysis, by luciferase assay or on the level of mutp53 target gene transcription.**
(DOC)
