# Supplementary figures and images for: Preferential Binding of Hot Spot Mutant p53 Proteins to Supercoiled DNA In Vitro and in Cells
Source: PLoS One. 2013 Mar 26;8(3):e59567. doi: 10.1371/journal.pone.0059567 (PMC3608670; doi:10.1371/journal.pone.0059567)

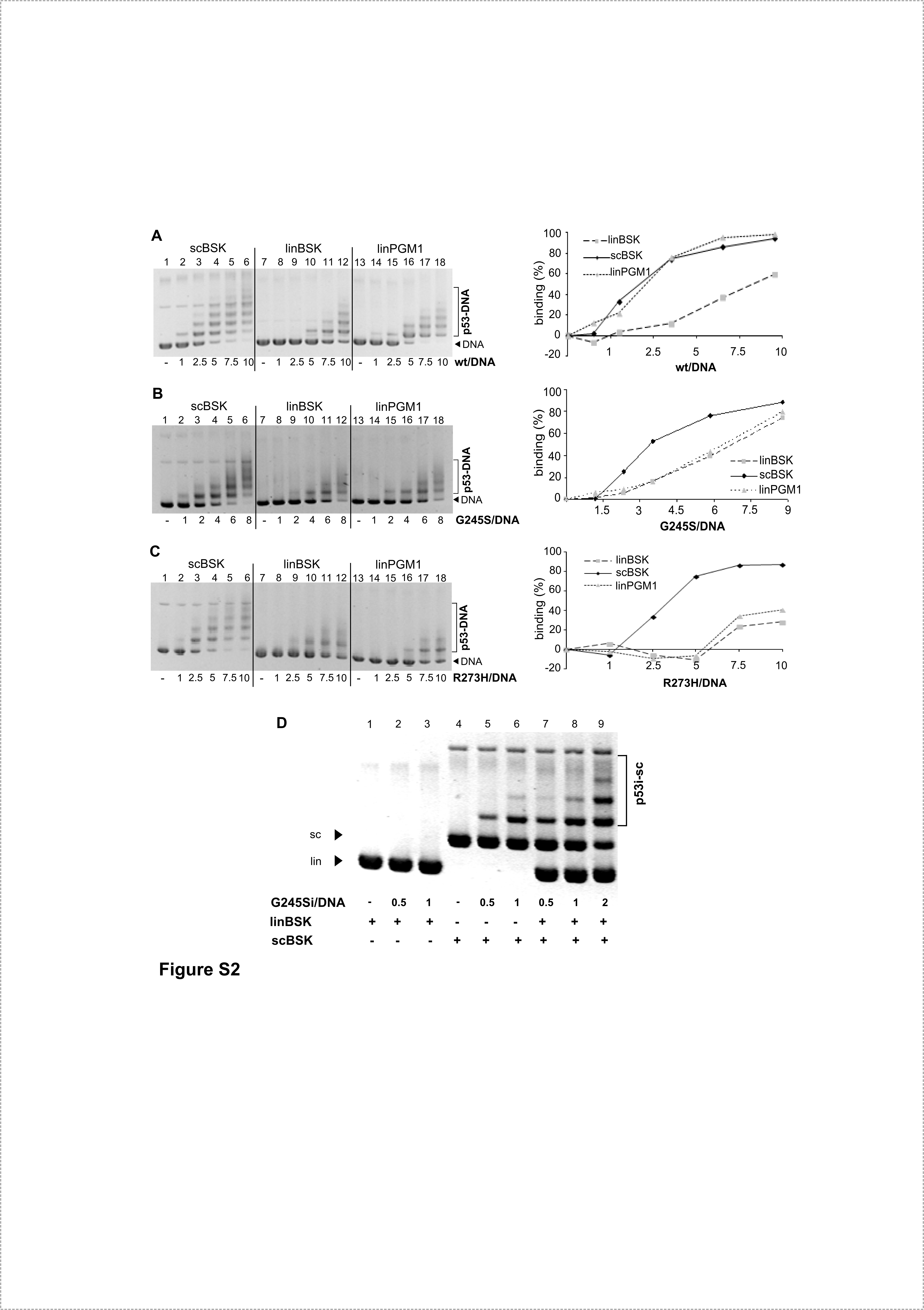

Supplement: Figure S2 — Comparison of mutp53 binding to scDNA and linDNA by EMSA and binding of baculoviral p53 protein G245Si to DNA (scDNA, linDNA, and their mix in sc/lin competition assays). A–C) Mutp53 proteins G245S, R273H and wtp53 were bound to scDNA (pBSK, 200 ng), linDNA (linBSK, 200 ng,) and to linDNA with p53CON sequence (linPGM1, 200 ng) in p53/DNA molar ratios as indicated in the figure for 20 min at 4°C before separation on 1% 0.33x TBE agarose gel. Graphs on the right were plotted on the basis of Et-Br stained agarose gels; free DNA substrates labeled with arrows were measured by densitometry. Graphs show the evaluation of p53-DNA binding as the dependence of % of bound DNA (axis y) on the amount of input of p53 proteins in the reaction (expressed by molar ratio p53/DNA, axis x). Bound DNA (%) were calculated as % of decrease of free DNA after binding of p53 in comparison to input DNAs (lanes 1,7 or 13 0% of bound DNA). D) Binding of mutp53i protein isolated from insect cells infected recombinant baculovirus. G245Si was incubated with 200 ng of scDNA (pBSK, lanes 5, 6), 200 ng linDNA (pBSK/SmaI, lanes 2, 3), both (mixture of 200 ng pBSK and 200 ng pBSK/SmaI, lanes 7–9) at molar ratios of p53/DNA 0.5–2, as indicated in the figure, for 20 min at 4°C before separation on 1.3% 0.33x TBE agarose gel. Binding of G245Si to DNA was detected by ethidium bromide staining (linDNA migrated faster than scDNA). (TIF) [file pone.0059567.s002.tif]

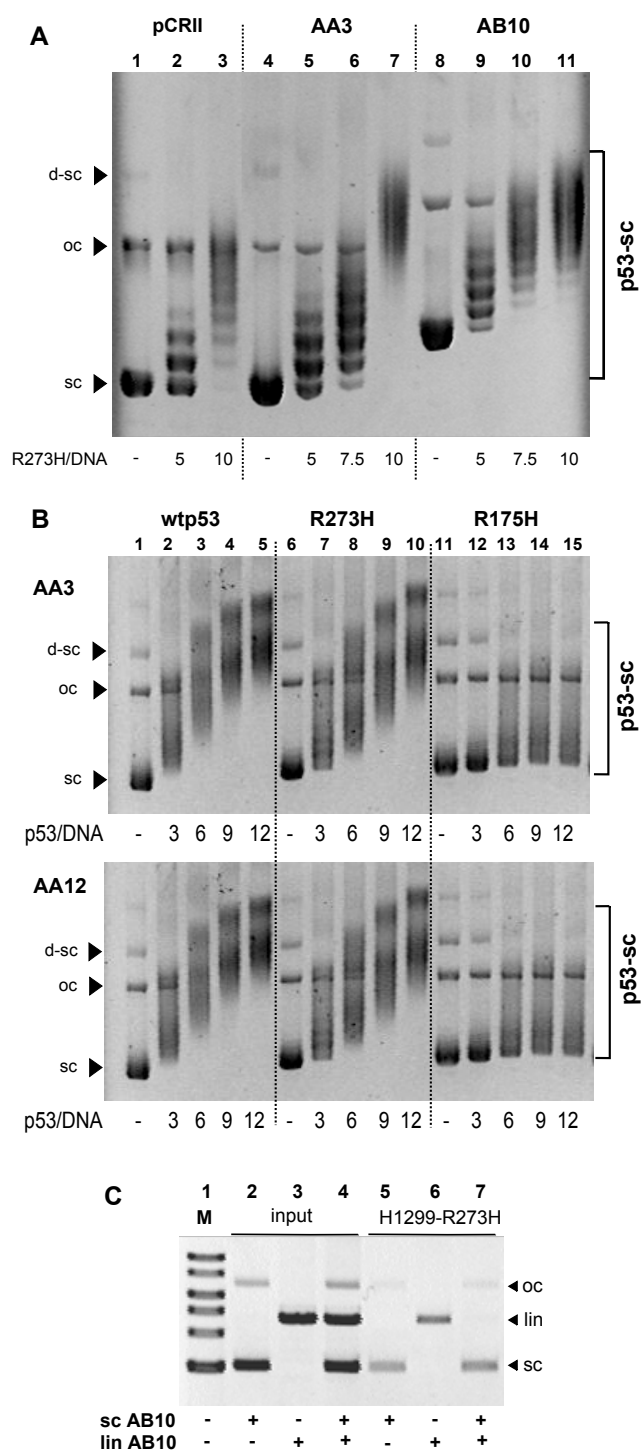

**Figure S5**

Supplement: Figure S5 — Mutant p53 recognition of mutp53BS (repetitive ChIP sequences) in scDNA. Repetitive ChIP sequences scAA3, scAB10, scAA12 were isolated as R273H binding sites (Tab. S1) from U251 cells [19] and cloned to pCRII vector. A) R273H binding to scpCRII (lanes 2–3), scAA3 (lanes 5–7), scAB10 (lanes 9–11) was compared at p53/DNA molar ratios of 5–10. B) Mutp53 proteins (R273H and R175H) and wtp53 bound scDNA form of scAA3 and scAA12 differently; binding to linear form was already shown [19]. Mutp53s and wtp53 were bound to scAA3 and AA12 at a molar ratio p53/DNA 3, 6, 9 and 12 and 150 mM KCl. P53-DNA binding and EMSA condition for A) and B) were the same as in Fig. 1. C) Binding of R273H from H1299 lysate to scAB10 (lane 5), linAB10 (lane 6) and to scAB10/linAB10 mixture (lane 7) by MBIP assays. Lanes 2–4 (scAB10, linAB10 and their mixture) were a control for input DNA (50 ng, 1/6 input DNA). After DO1 immunoprecipitation DNAs were analyzed on 1% TAE gel (scDNA migrates faster than lin or oc forms). The same conditions as in Fig. 5. (PDF) [file pone.0059567.s005.pdf]

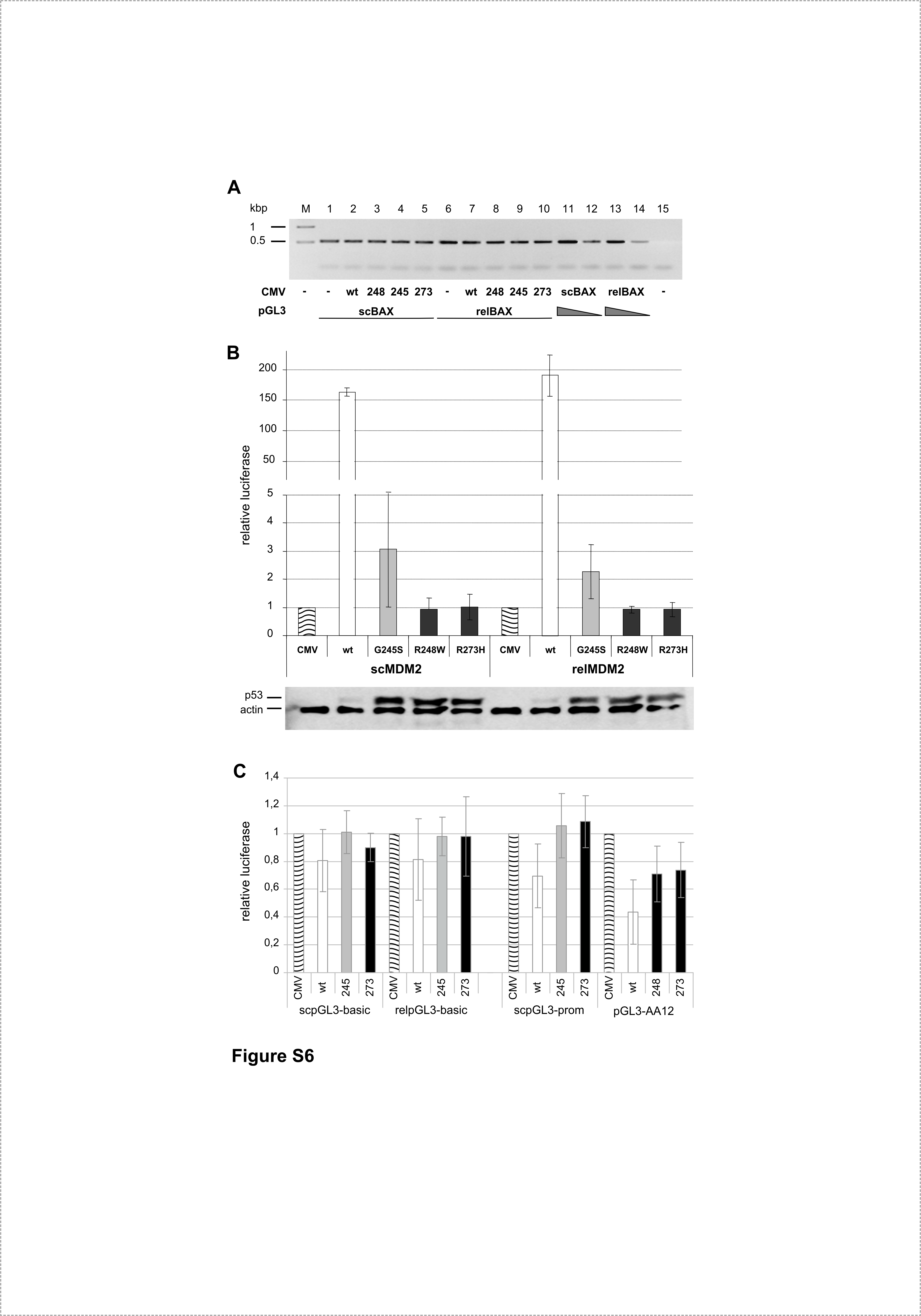

Supplement: Figure S6 — Influence of DNA topology on mutp53-driven trans-activation/repression measured by luciferase reporter assay. A) PCR control of DNA transfection for experiments on Fig. 7A. H1299 cells were transiently transfected with p53 expression plasmids (pCDNA) together with pGL3-BAX reporter plasmids (sc and rel form) and a reference plasmid with the renilla gene under control of the SV40 promoter. PCR analysis of isolated DNA from transfected cells was done with GL2 and RV3 primers. More details for transfection are on Fig. 7A. B, C) Dual Luciferase Assay showing influence of p53 proteins on gene promoters (pGL3-MDM2, pGL3-promoter, pGL3-basic, pGL3-AA12). H1299 cells were transiently transfected with p53 expression plasmids based on pCDNA3.1 or pCDNA3.1 alone (CMV) together with the reporter plasmids expressing the firefly luciferase gene under the transcriptional control of the indicated gene promoters and a reference plasmid with the renilla gene under control of the SV40 promoter. Experiments were analyzed 16–20 h post transfection and carried out in triplicates and at least three independent times; standards deviations are indicated. Representative western blot analysis of p53 and actin was performed using 50 µg of samples. B) Effect of MDM2 promoter topology on p53 transcriptional regulation. Both wtp53 and G245S activated scMDM2 and relMDM2. But R248W and R273H effects on sc and rel form of pGL3-MDM2 reporter were moderate. C) Effects of wtp53 and mutp53 on vectors pGL3-basic, pGL3-promoter and repetitive ChIP sequences pGL3-AA12. (TIF) [file pone.0059567.s006.tif]

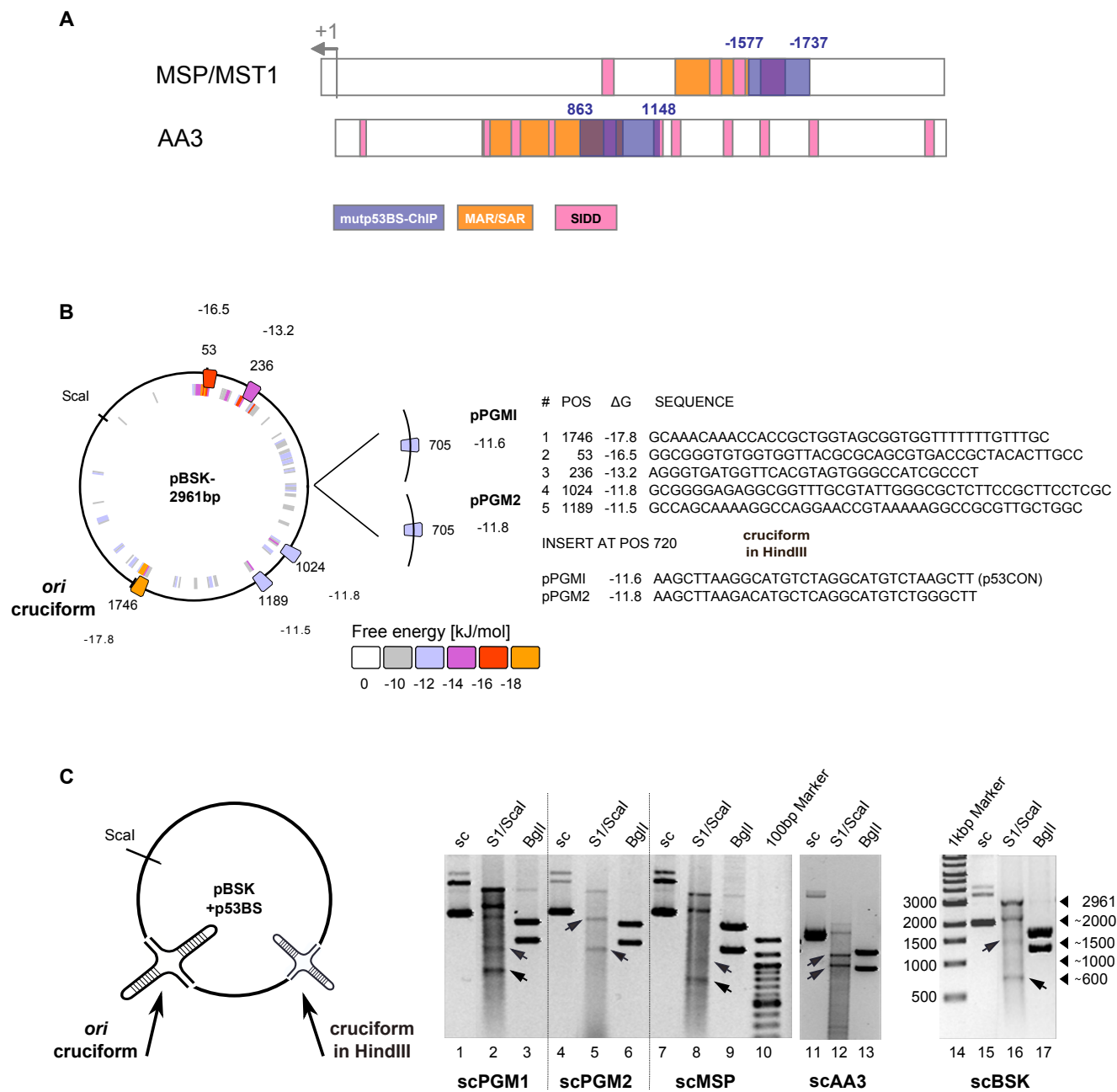

**Figure S8**

Supplement: Figure S8 — Non-B DNA structures in scDNA (pBSK, pPGM1, pMSP and pAA3) predicted by computational methods and detected by S1 treatment. A) In silico analysis of MSP/MST1 (chr3∶49,726,122-49,728,196) [28] and AA3 chip fragment (chr5∶81,887,426-81,889,425) [19] as DNA segments containing mutp53 binding sites (mutp53BS). Presence of putative S/MAR elements, regions with superhelical stress induced DNA destabilization (SIDD) and DNA triplexes (TD) was detected by the available tools: MAR-WIZ [64], a dynamic programming algorithm for identification of triplex-forming sequences [67] and SIDD [65]. The results obtained from these tools are summarized in diagrams showing the positions of mutp53BS (blue box), MAR/SAR (orange box) and regions with high SIDD (pick). B) The sequence of pBSK was analyzed using the UNAFold software package [89]. Whole-sequence folding prediction at 37°C yielded regions with predicted base-pairing (forced to a maximal distance of 64 bp). These regions were cut out and the free energy of structures formed by the used sequencesm was estimated using the program hybrid-ss from the UNAFold package. Position of the central base and the free energy of each structure are labeled on the outside of the plasmid and given with the sequences in the inserted table. In parallel, we scanned the sequence with a 48 bp sliding window and calculated the same parameters as above for each of the windows, obtaining a sliding numerical “folding potential” for every position on the plasmid. The value of the folding potential is shown grey-coded on the inside of the plasmid. Computational analysis predicted the presence of three regions with significant potential for hairpin formation, regions close to the origin and positions 1050 and 1750. Plasmids pPGM1 and pPGM2 contained inserts with a 26 bp palindromic sequence with a free energy estimated to be −11.6 and −11.8 kJ/mol, respectively. These values rank 4th compared with other similar sites in the pBSK-derived vectors. C) ScDNAs (p [file pone.0059567.s008.pdf]
