# Supplementary material for: Preferential Binding of Hot Spot Mutant p53 Proteins to Supercoiled DNA In Vitro and in Cells
Source: PLoS One. 2013 Mar 26;8(3):e59567. doi: 10.1371/journal.pone.0059567 (PMC3608670; doi:10.1371/journal.pone.0059567)
Supplement: Table S1 — Examples of mutant p53 binding sites identified by ChIP and confirmed by in vitro mup53 binding analysis, by luciferase assay or on the level of mutp53 target gene transcription. (DOC) [file pone.0059567.s009.doc]

**Table S1**

**Examples of mutant p53 binding sites identified by ChIP and confirmed by *in vitro* mup53 binding analysis, by luciferase assay or on the level of mutp53 target gene** transcription

| Gene/refs | Effect | Chromozom | ChIP-sequence | Non-B DNA prediction [15] |
| --- | --- | --- | --- | --- |
| MSP/MST1  [1] | downregulated | [chr3:49727698-49727858](http://www.genome.ucsc.edu/cgi-bin/hgTracks?hgsid=186811305&db=hg19&position=chr3:49727698-49727858&hgPcrResult=pack) | CTCACTGATGTGTAGCGGTGCTgtccaacacagcagccgtcatccacatg  aggttacttaaatttaaagttttaaaaattaacaattcgatttctcagtt  gccttagccacatttcaagagttcaatgaccacatgtGGCTAATGGTTAC  TCTGTTGGACA | 2x IR |
| MAP2K3  [2] | upregulated | [chr17:21187597+21187854](http://www.genome.ucsc.edu/cgi-bin/hgTracks?hgsid=186801553&db=hg19&position=chr17:21187597-21187854&hgPcrResult=pack) | CCTTTAGGGATCTCGGGTTTtttccagacgacgcacccaggagaacgcgc  ccgcgcatcgcacccgccggggctgcgggtctgggggtgccgggctgggc  ctgggcgcctgcagggagcagcgggctcagtgggtcccgggagtgggtgg  ggcttgcatgttgacgggcagcgcgaggggcgcggctttagtccaggggg  cggagcctgaccggacctgggtttttcgggcggctggcgGACTTGACAGA  GAGCGGGA | 2x G-QM |
| Id2  [3] | downregulated | [chr2:8821949+8822134](http://www.genome.ucsc.edu/cgi-bin/hgTracks?hgsid=186804197&db=hg19&position=chr2:8821949-8822134&hgPcrResult=pack) | GCACTTACTGTACTGTACTCTATttaccaccccagctgggctcgcgcccc  gcccaccccgcggggattggctgcgaacgcggaagaaccaagcccacgcc  ccgcgcccgcgcccaccaatggaagcgcccgctcgtcttgatagacgtgc  caccttccgccaatgggGACGAAGGGAAGCTCCAGC | 1x G-QM  1x MR |
| CD95/APO-1, FAS  [4] | downregulated | [chr10:90750911+90751155](http://www.genome.ucsc.edu/cgi-bin/hgTracks?hgsid=186804197&db=hg19&position=chr10:90750911-90751155&hgPcrResult=pack) | CCGCTGGGCAGGCGGGGCAGCTCCggcgctcctcggagaccactgcgctc  cacgttgaggtgggcgtggggtgcggacaggaattgaagcggaagtctgg  gaagctttagggtcgctggagggggaccccggttggagagaggagcggaa  ctcctggacaagccctgacaagccaagccaaaggtccgctccggcgcggg  tgggtgagtgcgcgccgccccGCGGGGGCGGGGAGAGAGCCTGCA | 2x IR |
| RLN2  (H2 relaxin)  [5] | upregulation | [chr9:5306549-5306891](http://www.genome.ucsc.edu/cgi-bin/hgTracks?hgsid=186804197&db=hg19&position=chr9:5306549-5306891&hgPcrResult=pack) | CTGCTACTTGTAAGAGACACTgggaaaccatgtctagcacttacagcctc  gaatgttcctgctgtcctacattcaaagacattactctgtacatattaac  cttcactattcagatgaactacaactggaactggatctaggatataggga  aatgaaggaaagggggcgttggaatccagtagacatgggttggaacccaa  actccagcaattgacagctgtatgcatggacatatttattgtttctcctt  aaaaagatttcctttttatttaaaggacttgaaatgccttttagttgtgc  ctgtaaagactgtgaatcttgcATTTGCCTGTGATCCTTAGCA | 1x IR |
| ASNS  [6] | upregulated | [chr7:97502414-97502621](http://www.genome.ucsc.edu/cgi-bin/hgTracks?hgsid=186811305&db=hg19&position=chr7:97502414-97502621&hgPcrResult=pack) | AATTTATTTCGGTGCTGatgtaattagtaaaatacgatttaagagtgtta  tgatactttttaaaaatcgtgatgtcttttcttcatcaaagagatactta  aaagcacataatcatcttgtggaggcaagttgacaaaatcctttttgaga  ttctaaaaggaatctttgtacttaaaatgtttaaggaccaCTGATGTATT  TTGGCTTC | 1x IR |
| GRO-1/CXCL1  [7] | upregulated | chr4:74734890-74735114 | TCAGAGTCCACAGGAGTTACTctgaagggcgaggcgcgggctgcatcagt  ggacccccacaccccacccgcaccccaagcgctccaccctgggggcgggg  ccgtcgccttccttccggactcgggatcgatctggaactccgggaatttc  cctggcccgggggctccgggctttccagccccaaccatgcataaaagggg  ttcgcGGATCTCGGAGAGCCACAGA | 1x G-QM |
| AA3  [8] |  | chr5:81888275-81888576 | TCATCCTCATCCTCATCCTTACCTTCCTCCTTCTCCTCCTCCTCCCTCTTA  TTATTATTCTTTCTTTTTTTTGGGGAAGTACTATCACAAACAAGTTTCATG  ATTAAAGAATGTTTTAAAACAAAGGGTATTTTTAAAAAGGATTTGAAAGAAAAATATAATAGTAAACACAAAAGAATCAAGTAATGGTGATATTTGGTGTTTCAATGAAGAGTGTAATTTTTAACTTGAATGATTTTTATCTCTCTGCTGCTCTCTCATGCATACTATATAAGCTGGACTGCAATTTAGCTTTAGTGATC | 1x IR  2x STR |
| AA12  [8] |  | chr7:152116576-152116908 | GATCAGAAATTATACTTCATTTGATACTTAATCACATGCAAAAAATCATTAGTTCCAGTTTTACAGTTGTTGCCGGTTATTTTTCTAACACAGATACAATGTCATACCTCTGTTTAACATCTTCAATGGCTCCCTATTGTTTACAGTTAATATTCAAAATGCCCAATTTACTTTCCCTTCCTCATTTCCTCCCATATTGGACTGTAGTCTCTTAAAAAGCAAAGGTCAGGGTCAGACACACCTGGATTCAAATCCTGACTCAATCACTTACAAGCTATATAAAAATTCATCTTTCATTTGTAAAACGTACTAATGATTCAACCCCATGTGATC | 1x STR |
| AB10  [8] |  | chr4:21548931-21549863 | GAAAAGAAGGAAAAAATCTTCAGATACAGAATTATGTTTAAATTTAATTA  AACTAAATATATACATATTTATGTCTGTACATTTATAGATTATATCTATA  TATATGACACATGCACTTTTTAAAAATAGCTCCTTTGGCAGAGTTTTGTG  TTAATATTTTCATTAAAAGAGGCTCTTATATGATAATATTGCTCACAATG  AAATGTAACAACTGTGTAGCTGGAAGCATTATCTCTCCTCTTTGGGCAAC  AGTCCCTGTAGTCTCTTGGTTATTCAGAATTTTAAAGGGACCATATTACC  CTATAAACTTAAGCATATGCACAAAACTGCTTTTGGGGAAGGCTACGATT  TCTAAACAATCTCAGATACATAAGGCCGAATGTCTGTTATCTGAAACGCT  TGAGACTACAAGTGTTTTAGATTTCAGATTTTTTTTGGATTTTGGAATAT  TTGCATACGTATTTGCAGATAGAAGCTATTTTATAGCTTGGGGATGAGTT  CCAAGCCTAAATCTAAAATTTGTTTATGTTTCATATATACCTTATACGTA  TAGCCTGAAGGTGACTTTATAAAATATTTTAATAATTTTGCGCCTGAAAC  AAAGTTTGTGTACACTGAACCATCAGAAAGCAAAGGTGTCCCAATCTCAA  CCACCCATGTGGACAATCTGTGGTTGTTTGGCATCATTATCATTCCTGAC  TCTGAATTTATATGCTATTAATAAGCATTTATTTTCTTACATTTATTCAC  ACATAAGTACTTAACAGTAAAATATATGGCATATTGTTAATGCAGTGAAA  ACATAATATGTTCAGGGTAACCAAGGAGCACAGCAGTGTCACCAGAATAC  CTGTTATCTGTTTAAAAACACCAGCAATCAAAAATGGCAAGTCTTCACTT  ATTCAAAAATGTTTATATTCTGCAGCATTTCGG | 5x IR |
| AA20  (FRMD5)  [8] | downregulated | chr15:44221155-44221509 | CTCTCCTGCCCATGGATATCGATGATGAGACAGAAAATGCAATTAACCTTAAATAGTTTAGGTCACTGGCCCAAGCTTGCTTACTGCTTGCTACCTCAGTCCCTTCCCTAAAGAATAGGCAAACCATAGAGAACCAGAACCAGGCGTTCCAGGGAGGAGCAAAAATAGGAATTATCCCACCCTCAGAGCTGCACAGTGATTCCAAGAGGCTCATGAATCATTCTGTGCACCATATAGAGAGAATAAATGGGTTGTACCTATATGCACTAGTATTTTCAAATATGTATAGGCACTGTTGTGGTTCATAAAATATAAATTTATTTTTTAAACGAGTGCTTAATAAGCTATTTTGATC | 1xDR  1x IR |

Non-B DNA motives: Inverted Repeat (IR), G-Quadruplex Motif (G-QM), Mirror Repeat (MR), Short Tandem Repeat (STR), Direct Repeat (DR) [9]

1. Zalcenstein A, Weisz L, Stambolsky P, Bar J, Rotter V, et al. (2006) Repression of the MSP/MST-1 gene contributes to the antiapoptotic gain of function of mutant p53. Oncogene 25: 359-369.

2. Gurtner A, Starace G, Norelli G, Piaggio G, Sacchi A, et al. (2010) Mutant p53-induced up-regulation of mitogen-activated protein kinase kinase 3 contributes to gain of function. J Biol Chem 285: 14160-14169.

3. Yan W, Liu G, Scoumanne A, Chen X (2008) Suppression of inhibitor of differentiation 2, a target of mutant p53, is required for gain-of-function mutations. Cancer Res 68: 6789-6796.

4. Zalcenstein A, Stambolsky P, Weisz L, Muller M, Wallach D, et al. (2003) Mutant p53 gain of function: repression of CD95(Fas/APO-1) gene expression by tumor-associated p53 mutants. Oncogene 22: 5667-5676.

5. Vinall RL, Tepper CG, Shi XB, Xue LA, Gandour-Edwards R, et al. (2006) The R273H p53 mutation can facilitate the androgen-independent growth of LNCaP by a mechanism that involves H2 relaxin and its cognate receptor LGR7. Oncogene 25: 2082-2093.

6. Scian MJ, Stagliano KE, Deb D, Ellis MA, Carchman EH, et al. (2004) Tumor-derived p53 mutants induce oncogenesis by transactivating growth-promoting genes. Oncogene 23: 4430-4443.

7. Yan W, Chen X (2009) Identification of GRO1 as a critical determinant for mutant p53 gain of function. J Biol Chem 284: 12178-12187.

8. Brazdova M, Quante T, Togel L, Walter K, Loscher C, et al. (2009) Modulation of gene expression in U251 glioblastoma cells by binding of mutant p53 R273H to intronic and intergenic sequences. Nucleic Acids Res 37: 1486-1400.

9. Cer RZ, Bruce KH, Mudunuri US, Yi M, Volfovsky N, et al. (2011) Non-B DB: a database of predicted non-B DNA-forming motifs in mammalian genomes. Nucleic Acids Res 39: D383-391.
